# Effect of the Promoting Resilience in Stress Management Intervention for Parents of Children With Cancer (PRISM-P): A Randomized Clinical Trial

**DOI:** 10.1001/jamanetworkopen.2019.11578

**Published:** 2019-09-18

**Authors:** Abby R. Rosenberg, Miranda C. Bradford, Courtney C. Junkins, Mallory Taylor, Chuan Zhou, Nicole Sherr, Erin Kross, J. Randall Curtis, Joyce P. Yi-Frazier

**Affiliations:** 1 Center for Clinical and Translational Research, Seattle Children’s Research Institute, Seattle, Washington; 2 Division of Hematology/Oncology, Department of Pediatrics, University of Washington School of Medicine, Seattle; 3 Division of Bioethics/Palliative Care, Department of Pediatrics, University of Washington School of Medicine, Seattle; 4 Cambia Palliative Care Center of Excellence, University of Washington, Seattle; 5 Children’s Core for Biomedical Statistics, Center for Clinical and Translational Research, Seattle Children’s Research Institute, Seattle, Washington; 6 Center for Child Health, Behavior, and Development, Seattle Children’s Research Institute, Seattle, Washington; 7 Division of General Pediatrics, Department of Pediatrics, University of Washington School of Medicine, Seattle; 8 Division of Pulmonary, Critical Care, and Sleep Medicine, Department of Medicine, University of Washington, Seattle

## Abstract

**Importance:**

Parents of children with serious illness, such as cancer, experience high stress and distress. Few parent-specific psychosocial interventions have been evaluated in randomized trials.

**Objective:**

To determine if individual- or group-based delivery of a novel intervention called Promoting Resilience in Stress Management for Parents (PRISM-P) improves parent-reported resilience compared with usual care.

**Design, Setting, and Participants:**

This parallel, phase 2 randomized clinical trial with enrollment from December 2016 through December 2018 and 3-month follow-up was conducted at Seattle Children’s Hospital. English-speaking parents or guardians of children who were 2 to 24 years old, who had received a diagnosis of a new malignant neoplasm 1 to 10 weeks prior to enrollment, and who were receiving cancer-directed therapy at Seattle Children’s Hospital were included. Parents were randomized 1:1:1 to the one-on-one or group PRISM-P intervention or to usual care. Data were analyzed in 2019 (primary analyses from January to March 2019; final analyses in July 2019).

**Interventions:**

The PRISM-P is a manualized, brief intervention targeting 4 skills: stress management, goal setting, cognitive reframing, and meaning making. For one-on-one delivery, skills were taught privately and in person for 30 to 60 minutes approximately every other week. For group delivery, the same skills were taught in a single session with at least 2 parents present.

**Main Outcomes and Measures:**

Participants completed patient-reported outcome surveys at enrollment and at 3 months. Linear regression modeling evaluated associations in the intention-to-treat population between each delivery format and the primary outcome (Connor-Davidson Resilience Scale scores, ranging from 0 to 40, with higher scores reflecting greater resilience) and secondary outcomes (benefit finding, social support, health-related quality of life, stress, and distress) at 3 months.

**Results:**

In total, 94 parents enrolled, were randomized to 1 of the 3 groups, and completed baseline surveys (32 parents in one-on-one sessions, 32 in group sessions, and 30 in usual care). Their median (interquartile range) ages were 35 to 38 (31-44) years across the 3 groups, and they were predominantly white, college-educated mothers. Their children had median (interquartile range) ages of 5 to 8 (3-14) years; slightly more than half of the children were boys, and the most common cancer type was leukemia or lymphoma. One-on-one PRISM-P delivery was significantly associated with improvement compared with usual care in parent-reported outcomes for resilience (β, 2.3; 95% CI, 0.1-4.6; *P* = .04) and for benefit finding (β, 0.5; 95% CI, 0.2-0.8; *P* = .001). No significant associations were detected between either platform and other parent-reported outcomes.

**Conclusions and Relevance:**

When delivered individually, PRISM-P was associated with improved parent-reported resilience and benefit finding. This scalable psychosocial intervention may help parents cope and find meaning after their child receives a diagnosis of a serious illness.

**Trial Registration:**

ClinicalTrials.gov identifier: NCT02998086

## Key Points

**Question:**

Does one-on-one delivery or group-based delivery of the Promoting Resilience in Stress Management for Parents (PRISM-P) intervention improve psychosocial outcomes, such as resilience and benefit finding, when compared with usual care among parents of children who receive a new diagnosis of cancer?

**Findings:**

This randomized clinical trial of 94 parents of children newly receiving a diagnosis of cancer found that compared with usual care, one-on-one delivery of the intervention was significantly associated with improved parent-reported resilience and benefit finding. These improvements were not observed with group delivery of the intervention.

**Meaning:**

This intervention may help parents cope and find meaning after their child has received a diagnosis of a serious illness.

## Teaser

This phase 2 randomized clinical trial compares the effects of individual- or group-based delivery of the novel intervention Promoting Resilience in Stress Management for Parents (PRISM-P) with the effects of usual care on improved self-reported resilience among parents of children receiving a new diagnosis of cancer.

## Introduction

Parents of children with serious illness experience significant stress and distress.^1,2^ Among parents of children with cancer, psychological distress is prevalent during the child’s treatment, and parents report higher anxiety, depression, and posttraumatic stress than population norms when treatment is over.^3,4,5,6^ These outcomes negatively affect surviving patients, siblings, and the family unit.^6^

To date, few interventions have specifically targeted the well-being of parents.^6^ Typical supports include referrals to professional psychosocial clinicians outside the pediatric hospital setting and only after parent distress becomes severe.^7^ Although parents endorse wanting support early in their child’s cancer experience, delivering interventions at this time is challenging; stress and adjustment to caregiving demands preclude participation, especially when programs demand caregivers’ time.^8^

Interventions targeting positive psychological resources are more promising.^9,10,11^ Resilience, an important construct, describes the process by which an individual harnesses resources to sustain psychological or physical well-being in the face of stress.^12^ Parents’ perceptions of their own resilience have been associated with clinically important outcomes, including psychological distress, health behaviors, and comfort communicating values to the medical team.^13^ Promoting personal resilience resources, including skills in stress management, problem solving, goal setting, cognitive restructuring, and meaning making, may reduce poor mental health outcomes and improve quality of life and health behaviors.^9,14,15,16,17,18^

Members of our group have previously developed a novel resilience intervention called Promoting Resilience in Stress Management (PRISM) for Adolescents and Young Adults (AYAs) with cancer.^19^ In a recent randomized clinical trial, compared with usual care (UC), PRISM was associated with higher patient-reported resilience, quality of life, hope, and benefit finding, and with lower psychological distress.^20,21^ Qualitative feedback from this and other studies by members of our group suggests that parents want a PRISM for themselves. Hence, a PRISM for parents (PRISM-P) was adapted and pilot tested.^11^

The objective of the present phase 2 randomized clinical trial was to determine the efficacy of 2 PRISM-P formats (group or one-on-one delivery) compared with usual psychosocial care among parents of children with cancer. Our primary aim was to determine if either intervention delivery format was associated with improved parent-reported resilience compared with UC 3 months following enrollment. We hypothesized that PRISM-P, with either format, would be associated with higher parent-reported resilience than UC. Our secondary objectives included explorations of the effect of each format on parent-reported benefit finding, hope, social support, health-related quality of life, stress, and psychological distress.

## Methods

### Design, Setting, and Participants

We conducted this phase 2, parallel, 1:1:1 randomized clinical trial at Seattle Children’s Hospital in Seattle, Washington, between December 2016 and December 2018 (trial protocol in Supplement 1). We followed the Consolidated Standards of Reporting Trials (CONSORT) reporting guideline for the trial conduct and results reporting.^22^ The Seattle Children’s Hospital Institutional Review Board approved this study. Eligible participants were English-speaking parents or legal guardians (hereafter called *parents*) of children who were 2 to 24 years old, received a diagnosis of a new malignant neoplasm 1 to 10 weeks prior to enrollment, were receiving cancer-directed therapy at Seattle Children’s Hospital, and had provided written informed consent (children aged ≥18 years), written assent (children aged 13-17 years), or verbal assent (children aged 7-12 years). Children younger than 7 years did not provide assent. All parent participants provided written informed consent. To avoid intrafamily correlation or data contamination, only 1 parent per family was eligible. If multiple caregivers in a single family were interested in participating, we asked them to identify the primary patient caregiver or the person who would most commonly be present at the ill child’s bedside. Each parent received a $25 gift card following each survey completion, for a total of $50 across the study.

### Recruitment and Randomization

Study staff (including N.S.) reviewed clinic rosters weekly to identify eligible families. Consecutive, eligible parents and their children were approached in outpatient clinics or inpatient wards. Following discussion of the study, parents and children provided consent or assent as described above.

**Figure 1.**
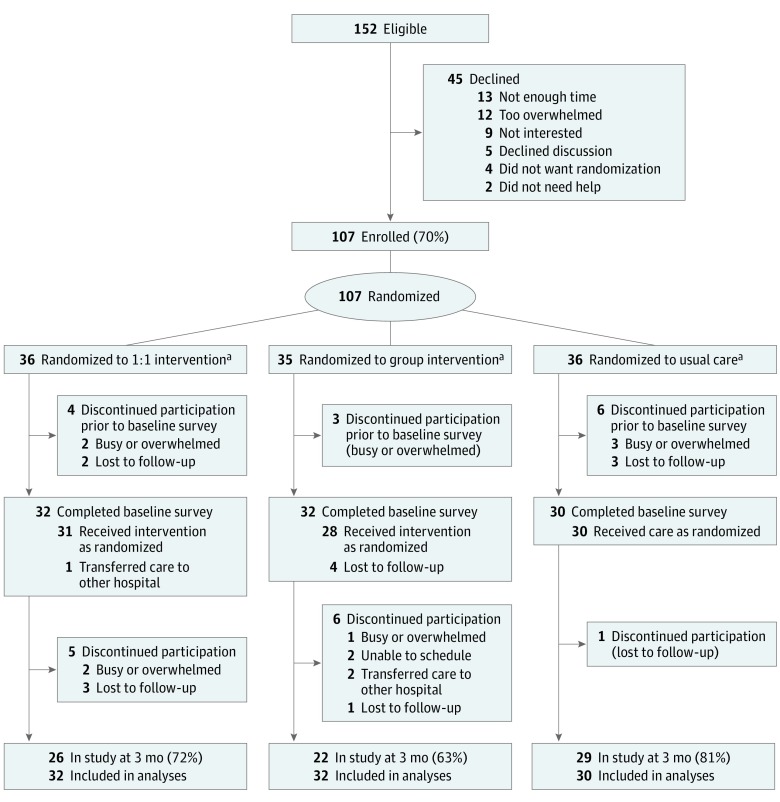
CONSORT Flow Diagram of Study Enrollment and Retention ^a^Participants were randomized into 1 of 3 groups: one-on-one (1:1) sessions, in which Promoting Resilience in Stress Management for Parents (PRISM-P) was delivered individually and privately to a single parent, with each of the 4 PRISM-P skills (to find out what these are, see the introductory paragraph of the text) taught approximately every other week; group sessions, in which PRISM-P was delivered to 2 or more parents at once, with all 4 skills learned in the same sitting; or usual care, in which no PRISM-P or other intervention was provided.

We continued enrollment until at least 22 parents in each arm completed the study (Figure 1). This strategy ensured comparisons between each intervention arm and UC. Our target sample size was determined from preliminary data suggesting that parent-reported resilience scores (primary outcome, measured with the 10-item Connor-Davidson Resilience Scale) were normally distributed, with a mean (SD) of 31.9 (6.3). Defining the minimal clinically important difference as half the SD,^23^ having 22 participants with complete data in each arm provided 80% power with a 2-sided α of .05 to detect the minimal clinically important difference in parent-reported resilience.

Parents were randomized 1:1:1 to UC alone, UC plus individual PRISM-P (one-on-one), or UC plus group PRISM-P (group). The randomization algorithm was constructed using permuted blocks with varying sizes. Owing to the nature of the intervention, we were unable to blind participants to randomization status.

### Usual Psychosocial Care

All participants received UC. At our center, this includes an assigned social worker who conducts a comprehensive assessment at the time of diagnosis and provides additional, ad hoc supportive care thereafter. Common assistance includes financial, housing, and other concrete resources. Although visits may provide a layer of professional psychosocial support, formal mental health support for parents (rather than for the child with cancer) is not routine. Parents judged by the clinical team to need professional counseling are referred to clinicians outside the hospital.

### The PRISM Intervention

We developed PRISM based on resilience and stress-and-coping theories, and successful positive psychology interventions described elsewhere.^11,19^ For the initial AYA-directed version, we modified cognitive behavioral therapy methods to create a preventative, brief, skills-based training program targeting 4 key resilience resources (eTable 1 in Supplement 2). These resources included (1) stress management, including relaxation and mindfulness exercises designed to recognize stressors and emotions without judgment; (2) goal-setting skills, including strategies to set “SMART” (specific, measurable, actionable, realistic, and time-dependent) goals and track forward progress; (3) cognitive reframing, including skills in recognizing negative self-talk and reappraising experiences realistically, if not optimistically; and (4) benefit finding, including exercises in identifying gratitude, meaning, and purpose despite adversity. Participants receive worksheets to practice skills between sessions, and intermittent, brief booster visits from study staff to practice individual skills.

For the parent-directed version (PRISM-P), we iteratively adapted the AYA program with semistructured parent interviews and pilot studies to test the PRISMA-P feasibility and acceptability.^11^ We kept the content and skills consistent across the AYA and parent versions to facilitate future implementation in family systems; we endeavored to create a program in which parents and children share similar language and skills to support one another. Both the AYA and parent PRISM are manualized (eg, standardized with protocols for training, delivery, and fidelity monitoring).^11,19^

We conducted 2 preliminary projects to explore delivery options of PRISM-P. First, we confirmed the feasibility and acceptability of one-on-one PRISM-P delivery.^11^ All 18 parent recipients (100%) endorsed PRISM-P and recommended it for other parents. Second, we held a half-day symposium in which 72 parents learned PRISM skills in small groups. Again, feedback from all participants (100%) suggested the program was valuable.

In the one-on-one arm of the present study, 4 separate sessions (1 session for each resource) were scheduled approximately every other week in conjunction with planned hospital admissions or outpatient clinic visits or by telephone, based on parent preference. Each session lasted no more than 60 minutes. Boosters were delivered in person or by phone.

In the group arm, all 4 sessions were conducted on the same day. We scheduled groups on Saturdays, approximately once every other month and endeavored to include 2 to 5 parents in each group. For instances in which a parent canceled or was unable to attend, they were invited to subsequent groups until they attended one or until 6 months after enrollment, whichever came first. Following the group session, participants were invited to sign up for an email list-serve with other members. Boosters were delivered via group email, and parents were invited to communicate electronically ad lib among themselves.

All PRISM-P sessions (one-on-one and group) were delivered by the same psychologist (C.C.J.). Per our manual, she received at least 8 hours of standardized training in PRISM-P scripts, including mock one-on-one sessions and group sessions, both with role playing. All sessions (one-on-one and group) were audio recorded. One of 5 randomly selected one-on-one sessions and all group sessions were scored for fidelity using a standardized tool.^19,24^

### Study Instruments

We requested demographic data in surveys and extracted child medical characteristics from the medical record. We asked all parents to complete a comprehensive survey composed of validated instruments with strong psychometric properties and demonstrated responsiveness at the time of enrollment (prior to any PRISM-P sessions, if relevant), and 3 months later. Surveys were offered by paper or online and delivered per parent preference. The domains evaluated by the parents and the study instruments used for those evaluations are given in the Box.^13,25,26,27,28,29,30,31,32^

Box. **Evaluated Domains and Study Instruments**

**Resilience.** The 10-item Connor-Davidson Resilience Scale assesses self-perceived resilience by querying an individual’s typical responses to adversity.^25^ Items are scored on a 5-point Likert scale; total scores range from 0 to 40, with higher scores reflecting greater resilience. Published mean (SD) scores are 31.8 (5.5) among healthy US adults and 30.0 (6.0) among parents of children with cancer.^13,25^
**Benefit finding.** The 14-item Benefit Finding Scale queries domains of personal growth, such as personal priorities, daily activities, and family.^26^ Total score is the mean of item scores, which range from 1 to 5, with higher scores suggesting higher benefit finding.
**Hope.** The 12-item Hope Scale measures the overall perception that one’s goals can be met.^27^ Total scores range from 8 to 64, and higher scores suggest greater hope.
**Social support.** The 19-item Medical Outcomes Study social support survey addresses emotional, informational, tangible, affectionate, and positive social interactions.^28^ Total score is the mean of item scores (range, 1-5), with higher scores suggesting better perceptions of social support.
**Health-related quality of life.** The Medical Outcomes Study 36-item short-form health survey evaluates physical functioning, body pain, physical and role limitations due to physical and emotional health problems, as well as emotional well-being, social function, fatigue, and general heath perceptions.^29^ Domain scores are computed algebraically and transformed to a scale of 0 to 100, with higher scores suggesting better health-related quality of life.
**Perceived stress.** The 10-item Perceived Stress Scale measures the degree to which life situations are appraised as stressful.^30^ Items are scored on a 5-point Likert scale; total scores range from 0 to 40, with higher scores reflecting higher stress.
**Psychological distress.** The 6-item Kessler Psychological Distress Scale screens for global psychological distress.^31,32^ Scores range from 0 to 24 points; higher scores reflect greater distress, with scores higher than 6 and higher than 12 suggesting moderate and high distress, respectively.

### Procedures

Following randomization, staff contacted each parent to share their assignment, schedule completion of the baseline survey, and, as applicable, PRISM-P sessions. When surveys were not returned within 1 week of their due date, staff contacted families by phone once weekly for 3 weeks. Those surveys not received within 12 weeks were considered missing.

### Outcomes

The primary outcome was the change in parent-reported resilience at 3 months relative to that at baseline. Secondary outcomes included changes in all other assessed instrument scores at 3 months.

### Statistical Analysis

In this intention-to-treat analysis, we analyzed and reported data as planned. Parents who were randomized and completed baseline surveys were included in all analyses regardless of whether they completed their assigned PRISM-P sessions or their follow-up surveys. Our primary aim was to examine associations between PRISM-P and changes in instrument scores between baseline and 3 months. We focused on changes because they are more clinically meaningful. However, given that all instruments had restricted ranges such that direction and magnitude of changes could be influenced by baseline values, score changes were regressed on study arms with baseline instrument values controlled for as covariates. We handled missing data with multiple imputation with chained equations in which missing variables were iteratively imputed fully conditional on other instruments at baseline and 3 months, and baseline characteristics.^33,34^ We generated 20 imputed data sets and reported the final pooled results across them. We defined minimal clinically important differences for linear instruments as half the SD of the mean pooled baseline scores.^23^ Per our a priori statistical plan, all testing was 2-sided and conducted at a statistical significance level of *P* < .05 without correction for multiple comparisons. We performed all statistical analyses, including multiple imputations, using Stata, version 15 (StataCorp). Data were analyzed in 2019 (primary analyses from January to March 2019; final analyses in July 2019).

## Results

Of 152 eligible parents, 107 (70%) enrolled and were randomized to one-on-one (n = 36), group (n = 35), or UC (n = 36), and 94 participants (one-on-one, n = 32 [89%]; group, n = 32 [91%]; and UC, n = 30 [83%]) completed baseline surveys (Figure 1). Participants across the 3 groups had a median (interquartile range [IQR]) age of 35 to 38 (31-44) years and were predominantly white, married mothers with at least some college education (Table 1). Their children with cancer across the 3 groups had a median (IQR) age of 5 to 8 (3-14) years; slightly more than half the children were boys, and the most common diagnosis was leukemia or lymphoma across the 3 groups.

**Table 1.** Participant and Child Characteristics at Baseline

Characteristic	No. (%) of Participants^a^		
	One-on-One Delivery of PRISM-P^b^	Group Delivery of PRISM-P^c^	Usual Care^d^
Parent participants, No.	32	32	30
Age, median (IQR), y	35 (31-41)	36 (32-44)	38 (34-44)
Relationship to patient			
Mother	26 (81)	25 (78)	22 (73)
Father	6 (19)	7 (22)	7 (23)
Other (adoptive grandmother)	0	0	1 (3)
Race			
White	18 (56)	23 (72)	22 (73)
Asian	3 (9)	3 (9)	6 (20)
African American	1 (3)	1 (3)	0
American Indian or Alaskan Native	0	3 (9)	0
Native Hawaiian or other Pacific Islander	2 (6)	1 (3)	2 (7)
Mixed/other	7 (22)	1 (3)	0
No answer	1 (3)	0	0
Hispanic or Latino ethnicity	4 (13)	1 (3)	1 (3)
First language English	30 (94)	29 (91)	28 (93)
Highest educational level			
<High school	4 (13)	0	0
High school	8 (25)	9 (28)	6 (20)
College/trade school	14 (44)	17 (53)	17 (57)
Graduate school	5 (16)	4 (13)	6 (20)
Other	1 (3)	2 (6)	1 (3)
Combined annual household income, $			
<24 999	5 (17)	7 (22)	4 (13)
25 000-49 999	3 (10)	3 (9)	3 (9)
50 000-99 000	9 (30)	12 (38)	9 (28)
≥100 000	13 (43)	10 (31)	10 (31)
Do not know or prefer not to answer	0	0	6 (19)
Partner status			
Married	22 (69)	24 (75)	21 (70)
Not married, living with partner	1 (3)	2 (6)	4 (13)
No partner, never married	4 (13)	4 (13)	1 (3)
Divorced, separated, widowed	5 (16)	2 (6)	4 (13)
No. of children at home			
Patient is only child	4 (13)	3 (9)	4 (13)
2	14 (44)	20 (63)	17 (57)
≥3	14 (44)	9 (28)	9 (30)
Children of parent participants			
Age, median (IQR), y	6 (3-13)	8 (4-14)	5 (3-11)
Sex			
Female	15 (47)	15 (47)	10 (33)
Male	17 (53)	17 (53)	20 (67)
Cancer			
Leukemia/lymphoma	18 (56)	15 (47)	18 (60)
CNS tumor	10 (31)	10 (32)	8 (27)
Non-CNS solid tumor	4 (13)	7 (22)	4 (13)

Abbreviations: CNS, central nervous system; IQR, interquartile range; PRISM-P, Promoting Resilience in Stress Management for Parents.

^a^ Participants were randomized to 1 of 3 groups.

^b^ PRISM-P was delivered individually and privately to a single parent, with each of the 4 skills (to find out what these are, see the introductory paragraph of the text) taught approximately every other week.

^c^ PRISM-P was delivered to 2 or more parents at once, with all 4 skills learned in the same sitting.

^d^ No PRISM-P or other intervention was provided.

Among the randomized parents, 31 parents in one-on-one (86% randomized and 97% with baseline surveys), 28 parents in group (80% randomized, 88% with baseline surveys), and 30 parents in UC (83% randomized, 100% with baseline surveys) received their intervention as assigned. In total, 26 parents in one-on-one (72% randomized, 81% with baseline surveys), 22 parents in group (63% randomized, 69% with baseline surveys), and 29 parents in UC (81% randomized, 97% with baseline surveys) completed 3-month surveys. There were no demographic differences between parents who received their assigned intervention vs those who did not. However, participants who may have had fewer immediate social and economic resources (eg, those who were not married and had low incomes) were overrepresented in the group who did not complete the 3-month surveys, and parents whose children had brain tumors did not complete 3-month surveys as often as other parents (eTable 2 in Supplement 2).

Among parents assigned to one-on-one PRISM-P, the median (IQR) time from enrollment to the first session was 18 (13-27) days, between sessions was 17 (14-27) days, and from enrollment to PRISM-P completion was 63 (57-77) days. Among parents in the group PRISM-P, median (IQR) time from enrollment to PRISM-P was 23 (12-47) days. The median (IQR) number of group participants was 2 (2-3). The median (IQR) time from enrollment to 3-month survey completion was similar across all groups: for parents in the one-on-one arm, it was 115 (98-125) days; in the group arm, it was 103 (97-109) days; and in the UC arm, it was 98 (93-108) days.

Raw survey scores for participants in each group at each time point are given in Table 2. Parents who did not complete the 3-month surveys had lower baseline instrument scores than other parents (eTable 3 in Supplement 2).

**Table 2.** Survey Scores for All Participants at Baseline and at 3 Months^a^

Survey Instrument	Mean (SD) Score												
	One-on-One Delivery of PRISM-P^b^			Group Delivery of PRISM-P^c^			Usual Care^d^			All		
	Baseline	3 mo	Change	Baseline	3 mo	Change	Baseline	3 mo	Change	Baseline	3 mo	Change
No. of participants	32	26	26	32	22	22	30	29	29	94	77	77
10-Item Connor-Davidson Resilience Scale	28 (6.0)	29 (6.0)	1 (5.3)	27 (6.0)	26 (5.0)	−1 (5.0)	30 (5.0)	28 (5.0)	−2 (3.5)	28 (6.0)	28 (5.0)	−1 (4.7)
Benefit Finding Scale	3.6 (0.9)	4.0 (0.6)	0.4 (0.6)	3.1 (0.9)	3.3 (0.7)	0.3 (0.6)	3.7 (0.9)	3.5 (0.8)	−0.1 (0.7)	3.4 (0.9)	3.6 (0.8)	0.2 (0.7)
Hope Scale, total score	51 (6.0)	52 (7.0)	0 (6.5)	48 (8.0)	48 (7.0)	−1 (4.9)	54 (4.0)	52 (6.0)	−2 (4.3)	51 (7.0)	51 (7.0)	−1 (5.4)
MOS social support survey, total score	4.2 (0.9)	4.1 (1.1)	−0.2 (1.0)	3.9 (0.9)	3.7 (1.1)	−0.3 (1.0)	4.2 (1.0)	4.0 (1.0)	−0.3 (1.1)	4.1 (0.9)	3.9 (1.1)	−0.3 (1.0)
HRQOL per MOS SF-36												
Physical functioning	91 (20.0)	93 (15.0)	−1 (9.0)	88 (19.0)	88 (19.0)	−3 (18.4)	88 (22.0)	87 (20.0)	−2 (28.6)	89 (20.0)	90 (18.0)	−2 (20.7)
Role limitations due to physical health	79 (36.0)	86 (33.0)	4 (37.3)	63 (44.0)	74 (39.0)	8 (38.2)	66 (44.0)	72 (40.0)	3 (37.5)	69 (41.0)	77 (37.0)	5 (37.2)
Role limitations due to emotional problems	59 (41.0)	77 (35.0)	20 (40.6)	54 (45.0)	56 (43.0)	−0 (50.4)	43 (41.0)	53 (48.0)	8 (44.2)	52 (43.0)	62 (43.0)	10 (45.0)
Energy and fatigue	34 (21.0)	41 (20.0)	5 (16.4)	37 (21.0)	39 (21.0)	−0 (17.4)	39 (20.0)	38 (20.0)	−1 (17.4)	37 (20.0)	39 (20.0)	1 (17.1)
Emotional well-being	58 (21.0)	66 (20.0)	7 (22.3)	57 (17.0)	60 (18.0)	3 (13.4)	56 (18.0)	58 (20.0)	3 (10.7)	57 (19.0)	61 (19.0)	4 (16.1)
Social functioning	59 (27.0)	72 (26.0)	10 (25.3)	63 (30.0)	66 (27.0)	2 (22.3)	52 (28.0)	63 (28.0)	11 (29.5)	58 (29.0)	67 (27.0)	8 (26.1)
Pain	77 (22.0)	86 (19.0)	4 (17.9)	77 (25.0)	70 (25.0)	−7 (17.6)	79 (19.0)	80 (21.0)	0 (15.3)	78 (22.0)	79 (22.0)	−1 (17.2)
General health	61 (20.0)	64 (18.0)	0 (15.2)	65 (16.0)	64 (19.0)	−1 (14.2)	69 (19.0)	64 (22.0)	−6 (12.3)	65 (19.0)	64 (20.0)	−2 (13.9)
Perceived Stress Scale	21 (6.0)	17 (6.0)	−4 (5.8)	22 (6.0)	20 (7.0)	−2 (5.9)	23 (6.0)	19 (6.0)	−4 (5.9)	22 (6.0)	19 (6.0)	−3 (5.9)
Kessler Psychological Distress Scale	9 (4.0)	6 (5.0)	−2 (3.8)	8 (4.0)	7 (5.0)	−2 (4.1)	10 (5.0)	9 (6.0)	−1 (3.6)	9 (4.0)	7 (5.0)	−2 (3.8)

Abbreviations: HRQOL, health-related quality of life; MOS, Medical Outcomes Study; PRISM-P, Promoting Resilience in Stress Management for Parents; SF-36, 36-Item Short Form Health Survey.

^a^ Participants were randomized to 1 of 3 groups.

^b^ PRISM-P was delivered individually and privately to a single parent, with each of the 4 skills (to find out what these are, see the introductory paragraph of the text) taught approximately every other week.

^c^ PRISM-P was delivered to 2 or more parents at once, with all 4 skills learned in the same sitting.

^d^ No PRISM-P or other intervention was provided.

### One-on-One PRISM-P Delivery Compared With UC

**Figure 2.**
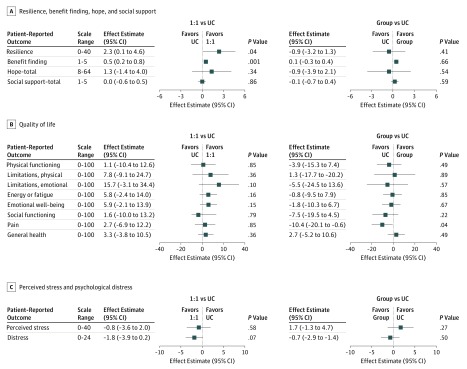
Mean Difference in Parent Outcome Score Change Associated With Promoting Resilience in Stress Management for Parents (PRISM-P) Intervention One-on-one (1:1) indicates PRISM-P delivered individually to a single parent, with each of the 4 PRISM-P skills (to find out what these are, see the introductory paragraph of the text) taught approximately every other week. Group indicates PRISM-P delivered to 2 or more parents at once, with all 4 skills learned in the same sitting. UC represents usual care, with no PRISM-P or other intervention. Squares indicate effect estimates; horizontal lines, 95% CIs from adjusted linear regression models after multiple imputation for missing 3-month scores.

Compared with parents who received UC, those who received one-on-one PRISM-P reported improved resilience (β, 2.3; 95% CI, 0.1-4.6; *P* = .04) and benefit finding (β, 0.5; 95% CI, 0.2-0.8; *P* = .001) (Figure 2). No other outcomes were significantly associated with PRISM-P.

### Group PRISM-P Delivery Compared With UC

There were no differences between parents who received UC and those who received the group PRISM-P (Figure 2).

## Discussion

Serious pediatric illness such as cancer is highly stressful to parents and caregivers. This stress may impair support for the ill child and well siblings and negatively affect the family unit.^6^ Recommendations for standard psychosocial care include early access to interventions to support parent coping.^6^ In this phase 2 randomized clinical trial, we compared 2 delivery formats of PRISM-P, a novel psychosocial intervention designed to build parental resilience, to usual psychosocial care. Our goal was to determine if 1 or both PRISM-P delivery formats helped parents of children with newly diagnosed cancer feel more resilient. We found that the one-on-one delivery was associated with improved parent-reported resilience (our primary outcome) and benefit finding (a secondary outcome). We identified no differences in other secondary outcomes, such as social support and health-related quality of life.

We chose self-perceived resilience as our primary outcome because it is strongly associated with individual well-being.^13,35,36,37,38^ Furthermore, PRISM-P targets resilience resources known to protect parents from stressors of cancer.^6,39,40,41,42,43,44^ Building new or strengthening existing resources may help them navigate the inevitable life changes accompanying serious pediatric illness. That one-on-one PRISM-P recipients felt more resilient and identified more benefit during their early cancer experience suggests that they may be buffered against the cumulative burdens of caregiving.

We did not observe the same improvement in resilience in the PRISM-P group delivery. There are several potential reasons for this finding, including that a gathering of parents who do not know one another may contribute to, rather than alleviate, stress, especially if they feel pressure to share personal experiences. Indeed, the 4 parents who declined to join our study because they did not want the randomization explicitly said that they did not want to be assigned to a group.

An additional limitation of the group was its intermittent scheduling. We were deliberately flexible to facilitate participation. Regardless, attrition was greatest in this randomization arm, perhaps because parents who were delayed in scheduling lost interest or found it difficult to attend. Although we had hoped to have at least 4 parents in each group, the median group size was only 2. Such a small number may have felt discouraging to those expecting more people or created additional pressures for parents to share their thoughts. We cannot determine if larger group sessions would have had similar or different results. Finally, the single delivery day may have undermined skill learning. In summary, a 1-day session with more than 1 parent present was not only less feasible, it also appeared less effective.

Neither PRISM-P delivery format was significantly associated with social support, health-related quality of life, stress, or distress. This finding may be because we were underpowered for these secondary outcomes. Although PRISM-P does not directly target social support and quality of life, we thought we would see more of an effect on stress and distress. Parent distress during pediatric cancer is important because it is associated with poor quality of life, physical comorbidities, and marital function.^3,45,46,47^ Parent distress typically spikes at the time of diagnosis and then stabilizes at a new normal 3 to 6 months later.^47,48,49^ Positive or negative psychosocial functioning in this later time frame predicts longer-term outcomes.^50^ Hence, we believe interventions with the potential to positively shape these trajectories are critical. Future iterations of PRISM-P will need to explore whether and how to directly focus on alleviating distress.

Providing psychosocial services to parents is difficult under the auspice of research or clinical service. To date, few interventions have been designed for parents, and fewer still have been successfully delivered early in the cancer experience.^6,9,10,51,52^ Clinical psychosocial professional staffing is often limited, and available personnel are necessarily focused on pediatric patients rather than on parents.^53^ Parents express concern about leaving their child’s bedside to focus on their own well-being, and many cannot engage in ongoing therapy.^54^ It is primarily for those reasons that early intervention delivery has been unsuccessful.^8^

We attempted to overcome these barriers by keeping PRISM-P brief (approximately 4 hours total). In our other studies, PRISM was delivered successfully by trained, nonclinical professionals, such as college graduates.^11,20,21^ Hence, it spared professional psychosocial staff for families with higher needs. In the present study, we limited potential variability in interventionist skill by having a single doctoral-level psychologist (C.C.J.) deliver all PRISM-P sessions (one-on-one and group). Although this strengthens the internal validity of our findings, it also limits them: whether we would see similar effects from lesser trained interventionists is unclear.

### Limitations

This study had several important limitations. First, we had a small sample size, which raises concerns about power stability of statistical modeling. Missing data compounded this problem both at baseline and at follow-up. Although our statistical methods were able to address the missing data for the 3-month outcome, we could not fully explore differences between parents who did and those who did not complete baseline survey data. That parents who were unmarried or had lower incomes at baseline were less likely to complete 3-month surveys. That they had lower baseline scores suggests that parents who may be in the most need of help were the least likely to remain in the study—an important limitation to the program—and that our efficacy results may be biased toward the null by these missing data because these are the parents who could reasonably be expected to improve the most from psychosocial interventions.

Second, similar to most parent intervention research, our sample was mostly white, English-speaking mothers at a large, well-resourced hospital. We enrolled only 1 parent per family and did not evaluate dyadic or intrafamily resilience. This limits generalizability, in particular to populations at higher risk for poor outcomes due to existing racial, ethnic, socioeconomic, or other health inequities. In addition, we lacked power and sample diversity to conduct relevant subgroup analyses.

Third, whereas the association between parent and child well-being is well established, we did not collect data regarding child health or the parent-child relationship. Thus, we could not determine whether or how PRISM-P delivery affected the children with cancer.

Fourth, how to optimize and operationalize PRISM-P remains unclear. We did not design this study to compare the efficacy of the 2 formats against each other; thus, we could not identify a clear winner. Our results suggested barriers and opportunities for both. Ideal formats may include options for parents, depending on whether they prefer group formats, and provision of additional resources to make the program feasible for parents who are single or economically disadvantaged.

## Conclusions

In summary, the PRISM-P intervention showed a positive effect on parent-reported resilience and benefit finding when delivered individually to parents of children with cancer. These findings underscore a critical goal in caregiver support: PRISM-P may help parents feel more resilient, which in turn may facilitate their continued ability to care for their child.

## Notes

Supplement 1. **Trial Protocol**

Supplement 2. **eTable 1.** PRISM Intervention Content Details **eTable 2.** Demographic Characteristics of Participants Who Did and Did Not Complete 3 Month Surveys **eTable 3.** Baseline Scores (Mean, SD) of Participants Who Did and Did Not Complete 3 Month Surveys

Supplement 3. **Data Sharing Statement**
