# Supplementary material for: Effect of the Promoting Resilience in Stress Management Intervention for Parents of Children With Cancer (PRISM-P): A Randomized Clinical Trial
Source: JAMA Netw Open. 2019 Sep 18;2(9):e1911578. doi: 10.1001/jamanetworkopen.2019.11578 (PMC6751761; doi:10.1001/jamanetworkopen.2019.11578)
Supplement: Supplement 1. — Trial Protocol [file jamanetwopen-2-e1911578-s001.pdf]

Seattle Children's
HOSPITAL • RESEARCH • FOUNDATION

PRISM
Promoting Resilience in Stress Management

Study Protocol

A Pilot Randomized Controlled Trial of the Promoting Resilience in Stress Management (PRISM) Intervention for Parents of Children with Cancer

Principal investigator:

Abby R. Rosenberg, MD, MS (Pediatrics, Seattle Children's Hospital)

Co-Investigators:

Michele Shaffer, PhD (Biostatistics, Seattle Children's Hospital)

Joyce Yi Frazier, PhD (CCTR, Seattle Children's Research Institute)

Research team:

Miranda Bradford, MS

Nicole Etsekson, MPH

Kaitlyn Fladeboe, MA

Stacy Garcia, BS

Lauren Huang, DNP, RN

Courtney Junkins, PsyD

Victoria Klein, BS

Jonathan Liaw-Gray, BS

Deepthi Nair, MS

Samantha Scott, BA

Krysta Shutske, PhD

Barbara Stansfeld, MS

Claire Wharton, BS

Study site: Seattle Children's Hospital

Protocol version: 06.14.2017

Seattle Children's
HOSPITAL • RESEARCH • FOUNDATION

PRISM
Promoting Resilience in Stress Management

Contents

List of Abbreviations ... 3

Research Synopsis ... 4

Background and Significance ... 5

Objectives ... 8

- Primary Objective ... 8
- Secondary Objectives ... 8

Study Design and Methodology ... 9

- Inclusion/Exclusion Criteria ... 9
- Participant Population, Screening and Enrollment ... 10
- Procedures ... 11

Analyses ... 15

Pitfalls and Alternatives ... 18

Timetable and Timelines ... 20

Informed Consent Process ... 21

Privacy and Confidentiality ... 21

Risk/Benefit ... 21

- Risk to participants ... 21
- Benefits to participants ... 22

Data Safety Monitoring ... 22

Conflict of Interest ... 22

Publication and Presentation ... 23

References ... 24

APPENDICES (separate attachments):

(1) RPCA
(2) Interview Guide
(3) Sample Handouts for participants
(4) PRISM Suicide Protocol
(5) PRISM Training Manual and Fidelity Scoring
    a. Scripts
    b. Role-playing scenarios for training sessions
    c. Fidelity Tool

List of Abbreviations

AYA: Adolescent and Young Adult

CRA: Clinical Research Associate

PRISM: Promoting Resilience in Stress Management

RCT: Randomized Controlled Trial

Research Synopsis

Study Title: A Pilot Randomized Controlled Trial of the Promoting Resilience in Stress Management (PRISM) Intervention for Parents of Children with Cancer

Study Population and Sample Size: Parents of children, adolescents and young adults with newly diagnosed cancer (n=75 parents)

Study Design: Pilot randomized controlled trial (RCT).

Primary Objective: To test the efficacy of two different versions of the "Promoting Resilience in Stress Management" (PRISM) among parents with cancer.

Primary Outcome: Change in parent-reported resilience (based on score of standardized Connor-Davidson Resilience Scale) at 3 months.

Secondary Outcomes:

(1) Parent-reported stress, hope, benefit-finding, social support, psychological distress, quality of life, and health behaviors at 3 and 6 months.
(2) Qualitative assessment of parent-reported goals at 3 and 6 months
(3) Ongoing parent-reported perceptions of usefulness, feasibility, and preferred format.

Study Duration: 2 years

Background and Significance

Parenting children with cancer is highly stressful and associated with psychological and sleep disturbances, impaired physical health, and financial hardship.[1] Parents of children with cancer are at high risk for debilitating psychological distress,[2] family dysfunction, and poor health behaviors.[3] These challenges have broad public health implications not only because they affect surviving patients and families for decades, but also because they impair survivors' and parents' abilities to contribute to society. Indeed, recent guidelines strongly recommend that parents of children with cancer receive early and ongoing psychosocial support in order to promote patient, parents, and whole-family wellbeing.[4,5] Evidence regarding feasibility and mechanisms of such supportive care interventions, however, is lacking.

Parents of children with cancer are an often understudied group; the impact of their well-being on that of the child may be overlooked. For this reason, rigorous research dedicated to promoting parent welfare and establishing normative parental support is warranted. In this regard, the promotion of positive psychological resources is particularly important because it may mitigate negative outcomes and support positive health-behaviors.[6] "Resilience" implies an ability to bounce back from adversity, and is evidenced by sustained emotional and physical well-being after significant stress.[7] The study of resilience in cancer is in the developmental phase;[8] existing research in other settings has lacked consensus of either definitions or outcomes indicative of resilience.[7] However, several personal resources are consistently associated with resilience in both adult and pediatric patients with cancer,[9,10] and parents of children with cancer.[3,8] These include stress-management, problem-solving and goal-setting skills, cognitive restructuring, and "benefit-finding," the ability to make meaning from adversity. We term this group of variables "resilience resources." Biobehavioral models suggest that resilience resources relate to long-term quality of life, health behaviors, neuroendocrine and immune function, and overall health and well-being;[11] hence, interventions designed to bolster these resources may improve both psychosocial and biological disease outcomes.[11]

Few interventions have obtained positive outcomes among pediatric cancer populations, and fewer still have suggested mechanisms to promote resilience resources.[12] Stress and coping theory,[13] however, provides an excellent platform for further study, highlighting three categories of resources that enable the maintenance of psychological well-being during serious illness: (1) dispositional factors (e.g., optimism); (2) situational factors (e.g., stress-management, goal-setting skills); and, (3) coping processes which create positive meaning (e.g., cognitive reframing). Among older adults with cancer, stress-management interventions show promise at the beginning,[14] middle,[15] and end of treatment,[16] and teaching meaning-making or benefit-finding may improve quality of life.[17] Among well adults, individual differences in goal-seeking and problem-solving have been associated with psychosocial well-being.[18] Likewise, among patients with chronic, non-malignant disease, teaching positive re-appraisal of stressors may reduce distress, and improve adherence[18] and quality of life.[19] Few interventions have focused on parents; however, problem-solving skills training has been associated with reduced distress and higher quality of life.[20] Furthermore, both childhood cancer survivors and parents have great potential for post-traumatic growth,[21,22] which may protect them from adverse psychosocial outcomes.

A practical challenge with positive psychology interventions has been the standard duration of cognitive behavioral therapy (CBT), typically 8-12 weekly 1-hour sessions. This is particularly challenging for parents of children with cancer. The average refusal rate for traditional CBT interventions in pediatric chronic disease settings is 37%; attrition rates are up to 32%.[23] Shorter skills-based interventions may be more successful.[24] Further, CBT is designed for patients with maladaptive coping, whereas parents may be helped to avoid maladaptive behaviors through preventive strategies. It follows that an effective intervention would briefly teach the skills to manage stress, set goals, re-appraise stressors and make-meaning.

In this pilot randomized trial, we will test and refine a novel intervention directed at parents' resilience resources. The successful completion of this project will fill important gaps in the scientific knowledge of pediatric psychosocial and palliative care oncology, translating to improved clinical support both during and after cancer. This research has the

potential to improve the emotional health, quality of life, and health-related behaviors of a group of cancer caregivers at very high risk for poor outcomes.

Preliminary Studies

Our central hypothesis is that promoting resilience resources will improve psychosocial outcomes among families facing pediatric cancer. We have defined "resilience resources" as several modifiable factors of ultimate emotional and physical well-being, including individual perceptions of stress, abilities to set goals and to find positive meaning in the face of adversity. We have followed a step-wise approach to investigate our hypothesis. First, we conducted a cross-sectional, mixed-methods study to explore the construct of resilience in parents of children with cancer. Qualitative findings affirmed our hypothesis, and directed the development a conceptual framework of resilience in pediatric cancer,[25] and a questionnaire comprised of validated instruments to measure parent-centered psychosocial outcomes [the "Resilience in Pediatric Cancer Assessment" (RPCA)].[3] Quantitative findings confirmed that parents with lower resilience resources had higher distress, lower social function, and poorer health behaviors.[3]

Second, we conducted the "Resilience in Adolescents and Young Adults with Cancer" study. In this longitudinal, multi-center, mixed-methods study, we conducted consecutive 1:1 semi-structured interviews with Adolescent and Young Adult (AYA) patients (ages 14-25) at the time of their diagnosis, 3- and 12-months later. Thematic analyses suggested that AYAs endorsed the need for strong resilience resources, but that they lacked the skills. Specifically, they stated stress-management and goal-setting skills, and "staying positive" and "making meaning" from adversity were essential to their well-being.[26-28] Together, these studies provided the rationale for the third step in our research program: the design and pilot study of a novel intervention to promote resilience resources: the "Promoting Resilience in Stress Management" (PRISM).[28,29]

The PRISM intervention was created based on stress and coping models (Figure 1),[13] our prior research, and successful interventions in the literature. The final design was refined with expert opinion and stake-holder interviews with patients, psychologists, social workers, and child life specialists. Details of the intervention are described below (Table 1). Briefly, its overall objective is to increase immediate resilience resources at times of high stress, thereby enabling coping, reducing distress and improving psychosocial outcomes. We completed a pilot study of the PRISM among AYAs and found the intervention to be feasible and valuable to both patients and their parents.[30] A Randomized Clinical Trial (RCT) of the AYA PRISM is ongoing and has enabled us to refine processes of enrollment, randomization, implementation, and data collection (PI: A. Rosenberg, Study SC-N114; SCH IRB# 15300).

Figure 1. The Role of Resilience Resources on Appraisal and Coping

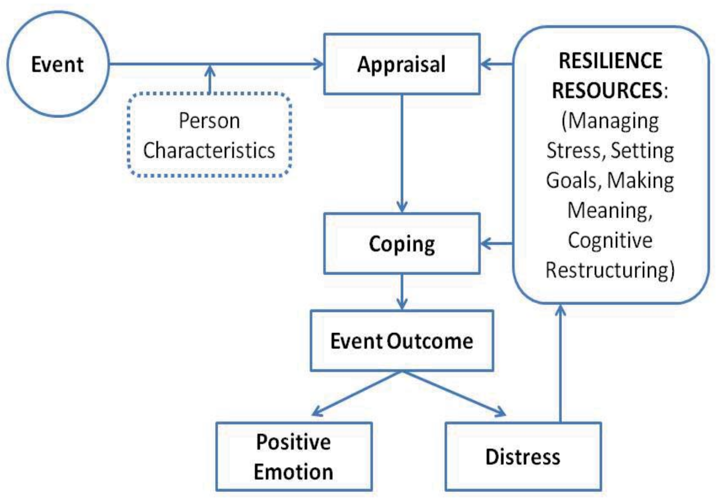

Adapted from Folkman S & Greer S; Psycho-Oncology 9:11-19 (2000)

Parents of all 12 AYA PRISM participants explicitly requested a similar intervention for themselves. Based on this experience, we adapted and piloted a parent-version of the intervention in two ways. First, we completed a pilot feasibility trial of a parent-version of the PRISM intervention (the "PRISM-P"). Like the original AYA-PRISM, the

parent design involves 4, 30-minute, 1:1 sessions approximately every other week. Thirteen (87%) of 15 approached parents enrolled, however, 3 (23%) dropped out of the study after reporting challenges with completion related to scheduling time away from their child. Several participants expressed the desire to include spouses and other caregivers in the same session, and almost all have requested to meet other parents in a similar situation. Among those who have continued to receive interventions, feedback was universally positive: *"[These are] things that everyone should have in their tool kit of life." –Patient's Mother*

Table 1. PRISM intervention content details.	
**Topic**	**Details**
1. Stress management	Breathing techniques, relaxation strategies, obtaining social support
2. Goal setting	Setting specific, realistic, desirable goals, planning for roadblocks
3. Cognitive restructuring	Recognizing negative self-talk, replacing these thoughts with positive/manageable ones
4. Benefit finding	Identifying benefits, purpose, meaning, or legacy from cancer experience

Based on this experience, our second iteration of the PRISM-P was a half-day symposium (hosted by the Seattle Children's Hospital Parent Advisory Committee) attended by over 70 individual parents. The PRISM-P was modified for group format: parents were seated at round-top tables of 8-10 and Dr. Rosenberg (PI) provided mini-lectures on: (a) resilience theory; (b) the 4 components of the PRISM (Table 2); and (c) examples of specific skills for each topic. In between these mini-lectures, parents participated in break-out groups to discuss their thoughts and experiences, and to practice skills. Trained moderators worked with each table. Feedback was universally positive; 92% of parents reported they learned new and helpful coping skills, and 98% reported the group format was helpful. Qualitative comments suggested high levels of satisfaction, and multiple parents in the community have since requested additional opportunities for participation.

In our prior observational and randomized trials, we have met or exceeded our target enrollment within the projected recruitment timeframe and anticipate we will do the same for this protocol. Our experiences suggest an opportunity to determine which PRISM-P format is most efficacious prior to larger-scale RCTs. While 1:1 sessions may provide more focused skills-training, the group format provides an additional layer of social support. Understanding the contribution of each format to parent-reported resilience and other psychosocial outcomes is critical to optimizing its success in future, larger, multi-site randomized controlled trials. This protocol will explore and compare effect sizes of each format compared to standard of care, and assess parent-reported usefulness and preferences.

Objectives

Primary Objective: To test the efficacy of the two different versions of PRISM-P intervention (individual- and group-based interventions compared to usual care) among parents of children with cancer.

Primary Outcome: Change in parent-reported resilience (based on score of standardized Connor-Davidson Resilience Scale) at 3 months.

Hypothesis: Each PRISM-P format will be associated with greater resilience (as measured by the 10-item Connor Davidson Resilience Scale, CDRISC), compared to usual care.

Secondary Objectives: To compare effect sizes of additional patient-centered outcomes across groups.

Secondary Outcomes:

(1) Parent-reported stress, hope, benefit-finding, social support, psychological distress, quality of life, and health behaviors at 3 and 6 months.
(2) Qualitative assessment of parent-reported goals at 3 and 6 months
(3) Ongoing parent-reported perceptions of usefulness, feasibility, and preferred format.

Hypotheses and expected findings: Patient-centered outcomes will vary by format of the intervention (e.g,. social support will be higher with the group format, whereas stress will be lower with the individual format).

Study Design and Methodology

Inclusion/Exclusion Criteria

Inclusion Criteria for parents:

1) Parents of children who:
    a. Are aged 2-24 years
    b. Have been diagnosed with new malignancy between 1-10 weeks prior
    c. Are scheduled to receive cancer-directed therapy at Seattle Children's Hospital
    d. Has provided written informed consent (child aged 18 years and older), written assent (child aged 13-17 years), verbal assent (child aged 7-12 years).
2) Able to speak and read English language
3) Cognitively able to participate in interactive interviews

Exclusion Criteria for parents:

1) Parent is < 18 years of age
2) Patient is <2 years of age, or >24 years of age.
3) Patient declines assent/consent (if >=7 years)
4) Parent is cognitively or physically unable to participate in interactive interview
5) Parent is unable to speak and read English language
6) Parent or child participated on prior PRISM intervention study

Inclusion Criteria for patients:

1) Child who:
    a. Are aged 2-24 years
    b. Have been diagnosed with a new malignancy between 1-10 weeks prior
    c. Are scheduled to receive cancer-directed therapy at Seattle Children's Hospital
    d. Has provided written informed consent (child aged 18 years and older), written assent (child aged 13-17 years), verbal assent (child aged 7-12 years)

Exclusion Criteria for patients:

1) Patient is <2 years of age, or >24 years of age
2) Patient declines assent/consent (if >= 7 years)
3) Patient participated on a prior PRISM intervention study

Participant Population, Screening and Enrollment

We will enroll consecutive parents of children with newly diagnosed cancer (N=75 parents, n=25 per arm). Parents will be eligible if they are English-speaking, their child is 2-24 years-old, has been diagnosed with new malignancy between 1 and 10 weeks prior to enrollment, and is scheduled to receive treatment at Seattle Children's Hospital (SCH). Two parents from the same family may enroll, they will be randomized together (by child) to an intervention arm. SCH treats approximately 250 new pediatric cancer patients each year, 80% of whom enroll on clinical trials, including survey- and interview-based studies. Conservative estimates suggest we will accrue our target sample in 9 months.

We will identify eligible parents through existing screening methods used in the hematology/oncology clinic. Specifically, trained and dedicated research associates will screen clinic patients for eligibility. We will request a waiver of HIPAA for screening purposes for this study, such that CRAs may identify potential and eligible families prior to their scheduled clinic visits. We will then contact patients' Seattle Children's primary oncology providers for all screened-eligible patients to verify appropriateness and to introduce the study. Study personnel may then initially contact parents to discuss the project while at a regularly scheduled outpatient visit or while inpatient. Study personnel may answer any questions the family has either on the phone or in person. Should the family prefer to speak in person, we will arrange a follow-up conversation regarding the study at the time of their clinic visit. This is a system that has been in place and worked successfully for over a decade at SCH.

All parents will provide written informed consent prior to enrollment. Also, given that we will identify parents through the child's medical record, and because some parents may disclose private information about their child during the sessions, we will ask the children of all participating to provide written consent (ages 18 and older), written assent (ages 13-17) or verbal assent (ages 7-12). Children younger than 7years will not be asked for assent.

Procedures

Overview: Following written informed consent, enrolled parents will be randomized (1:1:1) to one of the 3 arms: PRISM-P Individual (1:1), PRISM-P group, or standard of care (SOC, Table 2). Full RPCA surveys will be completed at the time of enrollment and then 3- and 6-months later for all groups. This time-point was selected to minimize heterogeneity of time constraints and optimize intervention completion (e.g., due to scheduling conflicts), and to enable parents in the combined arm to fully participate in both session-types. However, in order to assess immediate intervention effects, we will administer an additional, abbreviated survey querying only resilience at the 6-8 week-mark. This will likely fall immediately following the PRISM 4 session for the 1:1 intervention cohort and following completion of the group training for participants in the group intervention cohort. At 3 months, all participants will also be interviewed for their thoughts on the intervention structure, content, and perceptions of resilience. Due to the nature of the intervention, parents and staff will not be blinded. All surveys will be available by paper or online via the REDCap system, a secure HIPAA-compliant, high-quality data collection tool. Participants will receive a $25 gift-card following each full RPCA survey (at 0 weeks, 3 months, and 6 months; Table 2).

	**Table 2. Study timelines for pilot randomized trial of PRISM-P**								
Time from Enrollment	*0 weeks*	*2 weeks*	*3-4 weeks*	*5-6 weeks*	*6-8 weeks*	*6-8 weeks*	*Up to 6-months*	*3 months*	*6 months*
1:1 Intervention	RPCA	PRISM 1	PRISM 2	PRISM 3	PRISM 4	aRPCA,	Monthly “Boosters”	RPCA, interview	RPCA
Group Intervention	RPCA	Half-day symposium, PRISM sessions 1-4 (offered every other month)				aRPCA,	Monthly “Boosters”	RPCA, interview	RPCA
Standard of Care	RPCA	Standard psychosocial care				aRPCA,		RPCA, interview	RPCA
	RPCA: Resilience in Pediatric Cancer Assessment (See Table 1), aRPCA: abbreviated RPCA								

Randomization: The randomization algorithm will be constructed by the study statistician using a permuted blocks scheme with varying block sizes; only the statistician will be aware of the block sizes until completion of the study. A statistician independent of the study will prepare the final randomization list, which will be administered by a clinical research associate independent of the study using REDCap. As a quality control measure, a randomization log will be maintained by a separate CRA to track the participant ID, stratum, randomized assignment, and date of randomization.

The Resilience in Pediatric Cancer Assessment (RPCA) survey: Enrolled parents will complete the Resilience in Pediatric Cancer Assessment Instrument (RPCA) within 2 weeks of enrollment, and then 3-, and 6-months later (Tables 2 and 3). An abbreviated version of the survey, containing only the Connor-Davidson Resilience Scale (CD-RISC) will be used at the 6-8 week time-point. The complete RPCA has been previously developed and refined in prior studies at SCH (“Understanding Resilience in Parents of Children with Cancer,” IRB 13551) and “Resilience in Adolescents and Young Adults with Cancer,” IRB 14067). It includes a total of 162items and takes approximately 25-30 minutes to complete. The RPCA includes validated instruments to assess patient resilience, self-efficacy and goal-setting skills, benefit-finding, psychological distress, quality of life, risky behaviors and demographic variables:

a. CONNOR-DAVIDSON RESILIENCE SCALE (CD-RISC-10)

The Connor-Davidson Resilience Scale is a reliable and widely used instrument to measure inherent resiliency.[31] Questions revolve around personal problem-solving and approaches to adversity. The 10-item instrument has high internal consistency (Cronbach's alpha = 0.85), and has been used in diverse populations including adolescents, parents and cancer patients.[31,32] Correlative studies have evaluated the scale with other psychosocial measures such as psychological distress,[33] PTSD,[34] and social support.[35] It also has been used in pharmacologic and other intervention studies to model modifiable outcomes Each item consists of a 5-point Likert scale (scored from zero to four) for total of 40 points. The mean score among well US adults is 31.8, with higher scores reflecting greater resilience.

**Table 3. Instruments embedded in the Resilience in Pediatric Cancer Assessment (RPCA)**	
Skills targeted in PRISM	***Other patient-Centered Outcomes***
**A. Resilience**: Connor-Davidson Resilience Scale	**F. Psychological Distress**: Kessler-6
**B. Agency/Pathway**: Snyder Hope Scale	**G. Social Support**: Medical Outcomes Study Social Support Subscale
**C. Stress**: Perceived Stress Scale	**H. Quality of Life**: SF-36
**D. Goal Setting**: assessed qualitatively	**I. Health Behaviors**: Behavioral Risk Factor Surveillance Survey
**E. Benefit-Finding**: Benefit Finding Scale	

b. HOPE SCALE

The Hope Scale contains 8 hope items plus 4 "filler" questions, and measures "the overall perception that one's goals can be met."[36] The instrument distinguishes between the ability to generate a route to one's goals (termed "pathway" thoughts) and the ability to initiate and maintain the actions necessary to reach a goal (termed "agency" thoughts). Prior studies performed among AYA cancer patients have shown that high-hope individuals have improved psychosocial outcomes.[37] The instrument has been validated in both adult and pediatric settings and is scored on an 8-point Likert scale. Higher scores imply greater levels of hopeful thought patterns. The mean scores among well college students is 25 (SD 3.0) with higher scores indicating higher agency and pathway. Cronbach's alphas for the whole scale range from 0.74 to 0.84. In addition, the scale has demonstrated good test-retest reliability over time (correlations at 3-, 8-, and 10-week intervals are 0.85, 0.73, and 0.76, respectively (all $p<0.001$).

c. PERCEIVED STRESS SCALE

The perceived Stress Scale is designed to measure the degree to which life-situations are appraised as stressful.[38] The 10-item instrument has shown to correlate with multiple clinically relevant domains including life-event scores, depressive and physical symptomatology, utilization of heath services, social anxiety and health behaviors. Items include a 5-point Likert scale (scored from zero to four) for a total of 40 points, where higher scores indicate higher stress. Mean scores among well adults are 25.6 (SD 8.2) for women and 24.0 (SD 7.8) for men with cronbach's alpha=0.86.

d. QUALITATIVE GOALS

In order to assess learned skills of concrete goal-setting (in addition to perceived self-efficacy which is measured with the HOPE scale, above), the RPCA includes 3 open-ended questions regarding patients' goals for the next several months to years. For (a) the next few months, and (b) the next few years, each patient is asked to give an example a goal he/she hopes to accomplish, as well as how he/she expects to accomplish it. Qualitative responses will be de-identified and scored by 2 independent reviewers who are blinded to the patients'

randomization arm, as follows. First, reviewers will assign 1 point each for the goals that are (i) concrete; (ii) actionable; (iii) described with steps/pathways to completion; (iv) described with possible pitfalls and alternatives. (All of these items are taught in the PRISM goal-setting session). Inter-rater reliability will be tracked. When two reviewers agree, the numeric score (range 0 to 4) will be added to the database. If reviewers disagree, a third blinded reviewer will provide an additional score and the average of all three will be entered into the database. Second, reviewers will analyze all goals with open-ended coding and grounded-theory techniques[39] to ensure appropriate interpretation and translation of findings. Please see statistical analysis section for details.

e. BENEFIT FINDING SCALE
The Benefit Finding Scale (BFS) is a 14-item instrument (modified from an original, 20-item version) which queries domains of personal growth such as personal priorities, daily activities and family.[40] The scale has been validated among well adults and childhood cancer survivors[41,42] and has demonstrated high internal consistency (Cronbach's alpha scores of 0.86-0.95).[43] Only parent surveys will include this scale at the follow-up time points.

f. KESSLER-6 GENERAL PSYCHOLOGICAL DISTRESS SCALE (K6)
This 6-item scale measures "level of psychological distress experienced in the past month." It was developed for the US National Health Interview Survey,[44] and is currently being used in Canada, Australia and world-wide as part of the World Health Organization (WHO) world mental health initiative.[45] The instrument strongly discriminates between community cases and non-cases of Diagnostic and Statistical Manual of Mental Disorder (DSM)-IV psychiatric disorders such as serious emotional distress or serious mental illness (area under the curve [AUC] = 0.74-0.88).[46] It has been extensively cross-validated, including among adolescents.[47] Responses are scored on 5-point Likert scale, generating a range of zero to 24 points. Previous studies have shown that scores ≥ 7 are consistent with "high" distress and those ≥ 13 meet criteria for serious, or debilitating psychological distress.[48] The mean score among well US adults is approximately 3 and the prevalence of serious distress is 2-4%; however, our recent studies have shown that mean scores among parents of children with cancer may be significantly higher.[2]

g. MEDICAL OUTCOMES STUDY SOCIAL SUPPORT SUBSCALE (MOS-SS)
This 9-item multi-dimensional, social support survey is adapted from a longer 19-item version, originally developed for the Medical Outcomes Survey (MOS).[49] It addresses five, previously defined, functional dimensions of social support: emotional, informational, tangible, affectionate, and positive social interaction. Overall scores suggest level of functional support; for each sub-scale, simple algebraic sums are computed, and then raw scale scores are transformed into a scale of zero to 100. Higher scores suggest better perception of social support. The subscales have been found to be valid, reliable (Cronbach's alpha > 0.91), and stable over time. The Social Support MOS has been used in adult cancer patients and healthy adults.[50,51] Only parent surveys will include this instrument.

h. QUALITY OF LIFE: MEDICAL OUTCOMES STUDY RAND 36-ITEM HEALTH SURVEY (SF-36)
This 36 item questionnaire incorporates questions related to eight health concepts: physical functioning, body pain, limitations due to physical health problems, role limitations due to personal or emotional problems as well as emotional well-being and social functioning, energy, fatigue and general health perceptions. It also includes a single item that provides an indication of perceived change in health. It was adapted from longer instruments completed by patients participating in the Medical Outcomes Study (MOS).[52] Internal reliability ranges from alpha = 0.78 for the general health scale to 0.93 for the physical functioning scale.

i. HEALTH BEHAVIORS: BEHAVIORAL RISK FACTOR SURVEILLANCE SURVEY
The Behavioral Risk Factor Surveillance System (BRFSS), conducted by the Centers for Disease Control (CDC), is the world's largest continuous telephone survey. It covers civilian, non-institutionalized adults in all 50 states. In 2009, the survey collected data regarding health-related behavior from over 400,000 participants.[53] We have adapted 13 questions regarding behaviors such as sleep, tobacco use, and alcohol consumption. While formal scoring methods involve a complicated algorithm, population-based data and trends are available to the public on the CDC web-site and may be used as comparison to study-specific summary and descriptive statistics.[54]

j. ADDITIONAL QUESTIONS ABOUT THE CHILD'S CANCER EXPERIENCE AND PARENT DEMOGRAPHICS
In order to capture clinically meaningful variables such as patient diagnosis and parent-perceptions of communication, we adapted 15 additional questions from the Survey of Caring for Children with Cancer.[2] These and other demographic data (e.g., parent age, gender, race/ethnicity, level of education and, for parents, household income) will be self-reported.

To ensure data quality, a research associate (RA) will review RPCA surveys within 48 hours of their completion for missing fields and will call participants to clarify as warranted. All surveys will be available for completion in person, by paper, or online via the REDCap system, a secure HIPAA-compliant, high-quality data collection tool. We will schedule each participant for dedicated survey-completion appointment in tandem with other scheduled oncology visits. We will meet patients in person or send them an email invitation to complete their survey via REDcap. Those who fail to complete their surveys within 1 week will receive a phone call from the research associate. They will receive a $25 incentive following completion of each full survey.

Surveys may be completed in person or by phone (verbally), online using REDCap, or by paper-and-pencil. Participants will be offered all options. Participants will be given the survey prior to their first PRISM session and asked to complete it before the session begins. The abbreviated (aRCPA; CD-RISC instrument only) surveys will be administered at 6-8 weeks; follow-up surveys will be administered at 3- and 6-months following enrollment. If by chance the participant is on the experimental arm and has not completed all PRISM sessions by 6 months, we will still request the survey to be competed at 6 months, prior to completion of the study visits.

The Promoting Resilience in Stress Management (PRISM) intervention.

Enrolled patients randomized to the experimental arms will be invited to schedule and complete the PRISM intervention per their assigned arm (group vs 1:1). Details of the sessions are listed in Table 1. Briefly, session 1 ("Stress-management") focuses on mindfulness skills including deep breathing and relaxation techniques, and building awareness and acceptance of stressors. Session 2 ("Goal setting") teaches simple goal-setting skills (e.g., identifying realistic, concrete and actionable goals, planning steps towards their achievement, preparing for roadblocks and identifying alternative pathways). Session 3 ("Cognitive Restructuring") trains patients to recognize negative emotions and demoralizing self-talk and helps them develop skills to reframe these in a positive light. Session 4 ("Benefit Finding") focuses on finding meaning and/or benefit from difficult situations (including cancer).

- ***Individual (1:1) sessions***. Parents will schedule 4 separate sessions to be conducted approximately every 1-2 weeks (Table 3). Parents will also receive once monthly "booster" contacts until they reach the 6 month point from enrollment. Our experience suggests that advanced scheduling enables parents to plan and attend sessions, so these will be coordinated with planned inpatient-stays and/or clinic visits and added to parents' calendar of visits in our electronic scheduling system. The duration of each session will not exceed 60 minutes, and after the first visit is conducted in-person, follow-up sessions can be conducted either in-person, by phone, or by Skype. The Booster sessions will be brief (10-20 minute) opportunities to practice and review specific skills

(at the parent's discretion). All interventions will be administered by a trained non-clinical or clinical professional.[55] The PRISM has manualized training protocols. In our pilot, we successfully trained 4 interventionists who mastered delivery of the PRISM with high fidelity.[28] All sessions will be audio-recorded for fidelity. One of the PI's or coordinator supervisors will review the first 5 and then 1 of 4 randomly selected sessions for each interventionist. Fidelity will be scored using a standardized tool.[30] Interveners will receive feedback and approach will be refined if needed. We may also use the recordings to retain feedback from participants to improve the intervention, we will not keep any participant specific information.

- ***Group-based sessions***. We will schedule half-day long symposia to cover all 4 of the first PRISM sessions in one sitting. These will take place either in-person, by skype, or by phone. These will be scheduled in advance on one weekend-day or weekday evening every other month during the course of the study. We expect 4-6 parents will participate at each group workshop. These workshops will be led by and/or facilitated by the PIs or by trained non-clinical or clinical professionals. These sessions may be audio recorded for fidelity purposes. Fidelity will be scored using a standardized tool. Facilitators will receive feedback and approach will be refined if needed. We may also use the recordings to retain feedback from participants to improve the intervention, we will not keep any participant specific information. Light refreshments will be available for participants throughout the session. Following each session, parents will be asked privately if they wish to share their email with others in the group. For participants who willingly share their email, study staff will provide once monthly "booster" contact via email to the whole group, checking in and inviting the group to virtually share practiced skills and experiences. Email "booster" participation will be entirely voluntary; participants may share as much or as little as they like. Emails to the group will be monitored by study-staff and participants will be reminded not to share patient-identifying information. Text from emails will be cut and pasted into de-identified files for future evaluation of participant perspectives, strengths and limitations of the study. No identifying information will be saved. Participants may remove themselves from the group at any time. Since we are asking parents to travel to Seattle Children's on a day that may not align with when they are already here, participants who will be traveling more than 20 miles roundtrip may be compensated for their mileage driven (~ $0.54 per mile),

- ***Standard Psychosocial Care***. The Standard of psychosocial Care (SOC) at Seattle Children's includes an assigned social worker for each family. These professionals provide a breadth of services including administrative assistance with concrete needs (e.g., housing, insurance, financial aid, work-leave), as well as directed clinical support based on comprehensive needs-assessments conducted at the time of diagnosis. Ongoing support includes approximately weekly check-ins with families and additional referrals, as needed, for directed psychological or other support. Parents on all three arms of the study will receive this SOC.

Analyses

The <u>randomization</u> algorithm will be constructed by the study statistician using a permuted blocks scheme with varying block sizes. Our target <u>sample size</u> (n=75 parents) for this pilot study was based on estimates of patient accrual over a one-year period, projected intervention completion time, and anticipated duration of analyses. During the first 6 months of the study, approximately 125 new children with cancer will start their therapy at SCH. Based on prior experience of 80% enrollment rates and accounting for 10% attrition, we expect approximately 75 potential families to be eligible, enroll, and complete the study within the first year of funding. If attrition is greater than 10%, we will continue to enroll families so that at least 25 families complete the study in each therapy group.

Preliminary data from prior studies among parents of newly diagnosed AYAs indicate that 10-item Connor Davidson Resilience Scale (CDRISC) scores are normally distributed with mean score 31.9 (SD=6.3). We will have 80% power to

detect an effect size (change score) of 5.1 (Cohen's d=0.8) in each of the intervention arms versus SOC with a type 1 error rate (two-sided alpha) of 0.05 for each comparison. This is a moderate-to-large effect size, but our preliminary data of the PRISM among AYAs suggest this effect size or greater will be achievable. Power calculations for selected additional outcome measures are described in Table 4, assuming 80% power and a 5% type 1 error rate. Because this is a pilot, exploratory study, we will not adjust for multiple comparisons.

Table 4. Detectable Increase/Decrease in select outcome measures

**Selected Secondary Outcomes**	**Mean (SD)**	**Source**	**Change in outcome score**
Perceived Stress Scale	8.2 (3.5)	Published data [56]	2.8
Hope	42.7 (8.1)	Preliminary data	6.6
Psychological Distress (K6)	6.5 (3.9)	Preliminary data	3.2
Social Support (MOS-SS)	83.8 (15.1)	Preliminary data	12.2

Clinical variables will be abstracted from patients' medical records and include patient age, diagnosis, intensity of treatment in the 2-weeks prior to survey administration, and additional psychosocial needs/resources described in social work progress notes. These variables were selected based on prior evidence that child well-being and family psychosocial needs affect immediate psychosocial outcome metrics (e.g., psychological distress and quality of life).[57,58]

Statistical analyses will be performed with Stata 12 software. Demographic/clinical characteristics and RPCA items will be collected and summarized overall. We will use an intention-to-treat analysis plan to avoid confounding by non-random participant attrition. Hierarchical linear models[59] will be used to account for the multiple observations taken over time. Linear CD-RISC (resilience) score will be the outcome, and the PRISM intervention arm the predictor of interest. Contrasts of the hierarchical linear models, which are generalizations of two-sample t-tests, will examine if there is an increase in CD-RISC scores from baseline between the individual and combined therapy groups and the group-based and combined therapy groups at the primary time point of interest, 3 months. Similar analyses will examine secondary outcomes, modeled separately. In addition to the intention-to-treat analysis, we will conduct a per-protocol sensitivity analysis.

Parent-reported goals will be analyzed qualitatively. The RPCA queries current parent-goals with free-text items such as: "please given an example of a goal you hope to accomplish in the next month," and "how do you plan to accomplish this goal?" Responses will be de-identified and transcribed verbatim. Two independent and blinded reviewers will code each transcript in two ways: First, *a priori* scoring will include one point each for goals that are: (i) concrete; (ii) actionable; (iii) include steps to completion; (iv) include pitfalls and alternatives. When reviewers agree, the numeric score (0 to 4) will be added to the database. If reviewers disagree, a third blinded reviewer will provide an additional score and the average of all three will be used. Second, reviewers will analyze all goals with open-ended coding and content analyses[39] to ensure appropriate interpretation and translation of findings.

Parent-reported perceptions of usefulness, feasibility, preferred format, and mechanism of building resilience will be analyzed qualitatively. Our *a priori* philosophical assumptions are that some parents will prefer 1:1 versus group-based interventions, regardless of their randomization arm. We also expect that parent-reported resilience (and other psychosocial outcome instrument scores) will vary depending on parent preferences. For example, parents whose resilience relies more heavily on social support may report that the group-format is better-suited for their needs. Similarly, we anticipate that parent reported usefulness, feasibility, and satisfaction with intervention content will depend on their ongoing psychosocial needs and the child's illness state. The above-referenced clinical data will be used to contextualize parent qualitative responses.

In order to fully understand and optimize the impact of the intervention, we will therefore conduct qualitative interviews following the 3-month survey for all enrolled parents. Queries for parents on the intervention arms will focus

on the intervention itself; whereas queries for those on the SOC arm will be directed at perceived resilience and ongoing needs (Table 5).

Table 5. Interview Guide

CONTENT	PRISM INTERVENTION ARMS (1:1 and group)	SOC ARM
Intervention feedback	What did you think of the intervention?<br>What helped?<br>What should be different?<br>What did you think of the timing?<br>What did you think of the content?<br>What did you think of the materials (worksheets)?<br>Would you recommend this to another parent? (Why/Why not)?<br>As you may recall, we have two formats of this intervention (describe the one parent did NOT receive); what do you think of this other option? Would you have liked that better? If so, why? If not, why not? What do you think of a combination of the two (and what might that look like?)	As you may recall, this study tested two types of resilience interventions (one for individuals and one for groups). What do you think of each of these options? Which would have been better for you? Why? What do you think of a combination of the two (and what might that look like?)
Resilience	What makes you resilient?<br>How have you learned to be resilient during your child's treatment?<br>How have you handled adversity before?<br>Who supports you?<br>What worries you?<br>What could we do to help support you better during this difficult time?	What makes you resilient?<br>How have you learned to be resilient during your child's treatment?<br>How have you handled adversity before?<br>Who supports you?<br>What worries you?<br>What could we do to help support you better during this difficult time?

All interviews will be audio-recorded and transcribed verbatim. The study team will meet at least once monthly beginning at month 3 of the study in order to review parent transcript content and evaluate baseline assumptions. Early on, we will use an inductive approach to identify early themes in parent satisfaction and feasibility. Later, as more parents complete the study and provide their post-intervention RPCA scores and interviews, we will use more deductive methods to identify trends based on demographics, style, patient age, and social support. Reviewers will analyze all forms of data and conduct content analyses[39] to ensure appropriate interpretation and translation of findings. At least two members of the study staff will code transcripts independently, and meet monthly to identify emergent themes according to content analyses. All discrepancies will be explored with complete study team; mutual coding will be determined by consensus conversation. Study staff will relay results and emerging impressions with participants at successive interviews.

Merging of results: Following completion of quantitative and qualitative analyses, we will integrate and merge our findings by revisiting the PRISM-P. Potentially divergent findings include specific applications based on patient-age, modifications based on adaptation style, or combinations of multiple formats. If needed, we will re-format PRISM-P to better meet parent preferences and needs. Validation of results will be assessed through ongoing feedback during later interviews, as well as in future studies designed to test the efficacy of the refined PRISM-P intervention.

Pitfalls and Alternatives:

(1) Absence of merged intervention: For this pilot RCT, we chose to compare 2 different versions of the PRISM-P with standard of care. However, prior evidence in other settings suggests resilience is multi-dimensional and best optimized with combined individual and group-level support.[60] Indeed, a more effective intervention may be a merged format with both individual and group-based components. For this pilot RCT, we elected first to demonstrate effect sizes of each individual format while also collecting parent-feedback. These data will enable us to appropriately merge the interventions, if appropriate, for future studies.

(2) Duration of PRISM intervention: We deliberately selected a brief, skills-based intervention because the time involved in traditional CBT-based interventions is prohibitive to parents of children with cancer,[23] and stress and coping models suggest brief interventions can be successful.[13] PRISM pilot data suggest patients and families value this approach; however, it is possible that the selected intensity will be insufficient, in particular in the group-based format. Parent feedback following the intervention will assess this possibility, as well as the alternative, that the 1:1 intervention is still too long to be feasible.

(3) Accrual and attrition: Power calculations were based on prior patient accrual and attrition on clinical trials at our center; however, it is possible that parents will decline participation over time, or that they will be too worried about their child to engage in the study. Parents participating in 1:1 interventions may be less likely to complete the full intervention than those who have a single day group session. As we have done previously, we will incorporate incentives, advanced scheduling, and tracking measures to optimize participation. We will also utilize the child life team at our center to provide child care during sessions. In order to better understand the barriers to participation, we will also track non-identifiable demographic information for all eligible, but non- or dis-enrolled patients, including number of parents in the household and available social support. In addition, if needed, we will explore alternatives to provide respite for enrolled parents such that they can fully participate.

(4) Clinically meaningful results: the PRISM intervention targets self-reported resilience; however, clinically meaningful changes in CDRISC scores have not been well-defined. We powered our analyses based on anticipated accrual, but may have too few participants to detect subtle changes. Hence, this exploratory analysis will focus on effect sizes of the intervention on CDRISC, but also multiple other outcome measures. Results will inform the development of larger, phase III studies.

(5) Patient age range: Parents of children of different ages may have different perceptions of intervention feasibility; those of younger children may be less willing to leave the bedside. Likewise, parents of younger children versus adolescents may cite very different parenting challenges in group sessions. We will therefore track patient age as a factor of participation and intervention success for future studies and will use it as a covariate in our analyses.

(6) Intra-family correlation: Based on prior experience, we will allow both parents from a single family to enroll on this study. Such intra-family correlation may bias our results, especially if parents share with one another their skills and/or their impressions. However, we feel that this practical experience on a pilot study will inform us of the intervention strengths and limitations and further strengthen the development of future studies. We will not cluster under patients in this pilot study, but larger studies will need to address this feature of the study design with clustered analyses.

(7) Gender and Cultural diversity: Mothers tend to be the primary caregivers at the bedside and more commonly enroll in psychosocial research studies. Hence, we expect an uneven distribution of mothers and fathers in our study. We will explore confounding by sex and adjust analysis accordingly. Regarding culture and language differences, the PRISM-P is currently available in English only. This is because interventionists, to date, are only English speaking, and because fidelity and engagement assessments are done by (English-Speaking) investigators. We have not yet conducted the intervention with interpreter-services because we do not know if an additional (unfamiliar) person would affect parent

willingness to participate in PRISM sessions. Future studies will include non-English adaptations of the intervention, as well as additional qualitative studies to assess its cultural applicability and appropriateness.

(8) Normal trajectories of parent distress and resilience. Prior trajectories of parent distress and resilience have not been well-described, however, evidence suggests distress is highest at the time of diagnosis and subsequent stressors (e.g., surgeries, relapse).[4] We may see that parent resilience improves over time, regardless of the intervention. For these reasons, we will track additional clinical variables and incorporate these, as needed, into our analyses. Likewise, we will deliberately explore multiple outcome variables and qualitative impressions of resilience in order to identify time-trends and sensitive measures of parent well-being.

Timetable for development of work:

Academic Years 2016-2018												
	Q1	Q2	Q3	Q4	Q1	Q2	Q3	Q4	Q1	Q2	Q3	Q4
Human Subjects Approval, Study set-up, training, and initial Recruitment	■	■										
Enrollment			■	■	■	■						
Intervention sessions/follow-up (3 months total)				■	■	■	■	■	■			
Intervention re-design and fine-tuning based on qualitative feedback								■	■	■		
Manuscript development and dissemination of findings							■	■	■	■	■	■
Follow-up multi-site study design, funding applications							■	■	■	■	■	■

Future Plans:

Findings from this pilot randomized controlled trial will inform the development of larger (multi-site) clinical studies aimed to formally test the intervention, as well as direct future pilot studies (e.g., Spanish version of the intervention). Likewise, findings from the present study may be translated to other settings, impacting more broadly on the care of patients and families in other medical or stressful situations. This study will enable better family-centered care after serious illness and ultimately enable the positive development and well-being of pediatric patients with serious illness and their families.

Informed Consent Process

A waiver of consent will be requested for screening purposes only. All patients who screen eligible will provide signed informed consent/assent prior to enrollment.

The consent meeting between the CRA or PI and eligible participants (with patients if applicable) will include an explanation of the study in developmentally appropriate lay-language. All participants and parents will be provided an opportunity to read the consent/assent form, to ask questions about the study and have those questions answered by the research team member before deciding about study participation and signing the consent/assent form. Parental interest study participation will be obtained first, then patient consent/assent will be obtained for all patients 7 years-old and older (verbal assent for ages 7-12 years, written assent for those ages 13-17, and written consent for those 18 years and older). If a patient indicates that he or she does not want his or her parents to participate in the study, that non-assent will override the parent's permission and the parent will be recorded as a refusal. The research team member will redirect any parent who attempts to convince their child to participate in the study and remind them of their child's right as a potential research participant to refuse participation without coercion. The CRA will emphasize to all patients and parents in developmentally appropriate lay-language that being in the study is their choice, that they may choose not to participate or may change their mind at any time and it will not affect how their nurses or doctors care for them.

After signing informed consent/assent, participants will then be randomized and asked to complete the baseline RPCA survey. Those who are randomized to the intervention arms will also be invited to schedule their first PRISM session. Should patients or families change their minds and decline the study, the scheduled PRISM session(s) will be cancelled at no cost to the family.

This study includes children. Pediatric patients with serious illness are at risk for poor outcomes and may benefit from resilience-enhancing interventions in the future. We justify the inclusion of children in this project because the implementation of those interventions requires feasibility information and patient feedback. This study will provide those crucial data. Patients whose parents enroll in this study may, in fact, benefit from the intervention if their parents' coping skills improve and stress levels decline; however, at the time of consent, we will ensure that all patients/parents/families understand the objective of this study are to test the intervention such that it may be used prospectively in the future (see risks/benefits below).

Privacy and Confidentiality

All information collected for research purposes will be de-identified. Identifying information (names, addresses and phone numbers) will be used initially only to identify potential patients for approach. The only link between the Participant identifiers and their study identifier will be kept on a password protected database and in a locked filing cabinet. There are no patient identifiers collected and retained for research purposes. Feasibility data will represent only frequencies and percentages. Data will be stored in a locked office of the investigators and maintained for a minimum of three years after the completion of the study. An encrypted computer will be used for recording sessions completed over Skype.

Risk/Benefit

Potential Risks of Participation: The intervention ("Promoting Resilience in Stress Management", PRISM) may address sensitive matters in that it asks patients to identify stressors and negative thoughts. Participants may be prompted to think about the threat to their parenting roles posed by their child's cancer, as well as other difficult topics such as

emotional distress. The topics to be covered may provoke sadness, anxiety, depression, fear or doubt. Administrators of the intervention will be trained to recognize and query thoughts of self-harm or harm to others. If a parent expresses desire to self-harm or harm others during a study visit, an interaction with study team member, or written in a questionnaire, the parent will be referred immediately to their primary social work and medical teams. If a parent endorses severe psychological distress during a study visit or has a K6 score of 13 or above, the study PI will be notified and will decide if social work needs to be involved. As part of their informed consent process, patients will be made aware of this policy, as well as the fact that confidentiality may be broken in the case that providers see an immediate threat to the patient's or another's safety. No physical risks are expected to arise from the study.

Procedures to Minimize Risk: Subjects will be informed that they may refuse to answer any questions if they wish and may choose to stop participating at any time. The intervention has been adapted from similar tools used among cancer and diabetes patients, as well as other AYA and adult populations. No previous adverse events have been reported form previous investigations. If an event were to occur in this study, we would try to determine if there were a link between the questions and the adverse reaction and determine if any modification of the survey instrument would be advised. As above, subjects' responses will be monitored carefully and prompt referrals made to the appropriate mental health professional if warranted.

Benefits: We hypothesize that parents who receive the intervention will have diminished psychological distress and greater quality of life. However, there may be no direct benefit for participating in this study if our hypotheses are wrong. More broadly, information gained from this study may heighten the understanding of parents' cancer experience and elucidate strategies that foster resilience and promote better quality of life in this group. These strategies could be extended to the care of other parents facing non-cancer-related life-threatening illness in their children. This research has the potential to contribute to the research base concerning the promotion of optimal quality of life and mental wellness for all pediatric patients and families.

Alternatives: Participants may opt not to participate in the research. Their care will not be affected in any way should they decline participation.

Data Safety Monitoring

As above, a research associate will report immediate threats to participants' or others' safety to the PI within 24 hours of awareness. In cases of concern for participant or others' safety, immediate referrals will be made to the PI, and the patients' primary medical and social work teams for in-person evaluation or referral to the appropriate mental health professional if warranted. After hours, the PI and on-call providers from the medical teams will be notified. All concerns for participant or other person's safety will be reported as an adverse event (AE) to the IRB within 1 week. In addition, the PI will review the potential risks and reported findings at annual renewal. While this is not a therapeutic trial and we do not anticipated medical complications, we will nevertheless notify the IRB of all participant deaths. The study will be suspended for review if 2 patients or parents report threat to themselves or others.

Conflict of Interest

None of the investigators has any conflict of interest.

Publication and Presentation

Study results will be published in peer-reviewed journals and/or presented at professional meetings.

Data and/or Sample Sharing

Data will not be shared outside the group of investigators conducting the study but will be fully shared during and after the study with investigators in the group. De-identified study data will be banked indefinitely for future use by the group of investigators conducting the study and access will be controlled by the PI's. Future studies will formally test the intervention once its feasibility is confirmed. Should the intervention be effective, it will be made publically available for use by the broader medical communities caring for children with serious illness.

References

1. Northouse L, Williams AL, Given B, McCorkle R. Psychosocial care for family caregivers of patients with cancer. *J Clin Oncol.* 2012;30(11):1227-1234.
2. Rosenberg AR, Dussel V, Kang T, et al. Psychological Distress in Parents of Children With Advanced Cancer. *JAMA Pediatr.* 2013:1-7.
3. Rosenberg AR, Wolfe J, Bradford MC, et al. Resilience and psychosocial outcomes in parents of children with cancer. *Pediatric blood & cancer.* 2014;61(3):552-557.
4. Kearney JA, Salley CG, Muriel AC. Standards of Psychosocial Care for Parents of Children With Cancer. *Pediatric blood & cancer.* 2015;62 Suppl 5:S632-683.
5. Jacobsen PB, Holland JC, Steensma DP. Caring for the whole patient: the science of psychosocial care. *J Clin Oncol.* 2012;30:1151-1153.
6. Folkman S. Positive psychological states and coping with severe stress. *Soc Sci Med.* 1997;45(8):1207-1221.
7. Bonanno GA, Westphal M, Mancini AD. Resilience to loss and potential trauma. *Annu Rev Clin Psychol.* 2011;7:511-535.
8. Rosenberg AR, Baker KS, Syrjala KL, Back AL, Wolfe J. Promoting Resilience among Parents and Caregivers of Children with Cancer. *J Palliat Med.* 2013.
9. Molina M, Yi, J., Martinez-Gutierrez, J, Reding, KR, Yi-Frazier, JP, Rosenberg, AR. Resilience across the cancer continuum: a diversity of perspectives. *Clinical Journal of Oncology Nursing. In press,* 2013.
10. Rosenberg A, Yi-Frazier JP, Wharton C, Gordon K, Kroon L, Jones B. Contributors and Inhibitors of Resilience among Adolescents and Young Adults with Cancer. *J Adol Young Adult Oncol.* 2014.
11. Andersen BL, Thornton LM, Shapiro CL, et al. Biobehavioral, immune, and health benefits following recurrence for psychological intervention participants. *Clin Cancer Res.* 2010;16(12):3270-3278.
12. Haase JE. The adolescent resilience model as a guide to interventions. *J Pediatr Oncol Nurs.* 2004;21(5):289-299; discussion 300-284.
13. Folkman S, Greer S. Promoting psychological well-being in the face of serious illness: when theory, research and practice inform each other. *Psychooncology.* 2000;9(1):11-19.
14. Carpenter KM, Stoner SA, Schmitz K, McGregor BA, Doorenbos AZ. An online stress management workbook for breast cancer. *J Behav Med.* 2012.
15. Carver CS. Enhancing adaptation during treatment and the role of individual differences. *Cancer.* 2005;104(11 Suppl):2602-2607.
16. McGregor BA, Antoni MH, Boyers A, Alferi SM, Blomberg BB, Carver CS. Cognitive-behavioral stress management increases benefit finding and immune function among women with early-stage breast cancer. *J Psychosom Res.* 2004;56(1):1-8.
17. Henry M, Cohen SR, Lee V, et al. The Meaning-Making intervention (MMi) appears to increase meaning in life in advanced ovarian cancer: a randomized controlled pilot study. *Psychooncology.* 2010;19(12):1340-1347.
18. McCarty CA, Violette HD, McCauley E. Feasibility of the positive thoughts and actions prevention program for middle schoolers at risk for depression. *Depress Res Treat.* 2011;2011:241386.
19. van der Lee ML, Garssen B. Mindfulness-based cognitive therapy reduces chronic cancer-related fatigue: a treatment study. *Psychooncology.* 2012;21(3):264-272.
20. Sahler OJ, Dolgin MJ, Phipps S, et al. Specificity of problem-solving skills training in mothers of children newly diagnosed with cancer: results of a multisite randomized clinical trial. *Journal of clinical oncology : official journal of the American Society of Clinical Oncology.* 2013;31(10):1329-1335.
21. Zebrack BJ, Stuber ML, Meeske KA, et al. Perceived positive impact of cancer among long-term survivors of childhood cancer: a report from the childhood cancer survivor study. *Psychooncology.* 2012;21(6):630-639.
22. Barakat LP, Alderfer MA, Kazak AE. Posttraumatic growth in adolescent survivors of cancer and their mothers and fathers. *J Pediatr Psychol.* 2006;31(4):413-419.
23. Karlson CW, Rapoff MA. Attrition in randomized controlled trials for pediatric chronic conditions. *J Pediatr Psychol.* 2009;34(7):782-793.
24. Steinhardt M, Dolbier C. Evaluation of a resilience intervention to enhance coping strategies and protective factors and decrease symptomatology. *J Am Coll Health.* 2008;56(4):445-453.

25. Rosenberg AR, Baker KS, Syrjala KL, Back AL, Wolfe J. Promoting resilience among parents and caregivers of children with cancer. *Journal of palliative medicine.* 2013;16(6):645-652.
26. Rosenberg AR, Wharton C, Crouch JD, Wolfe J, Syrjala K, Baker KS. Promoting Resilience in Adolescents and Young Adults with Cancer. Poster Presentation of Abstract presented at CureSearch Annual Research Symposium; October 15-16, 2013, 2013; Washington, D.C.
27. Rosenberg A, Yi-Frazier JP, Wharton C, Gordon K, Kroon L, Jones B. Contributors and Inhibitors of Resilienc among Adolescents and Young Adults with Cancer. 2014, manuscript submitted.
28. Rosenberg AR, Yi-Frazier JP, Eaton L, Wharton C, Cochrane K, Pihoker C, Baker KS, McCauley E. Promoting Resilience in Stress Management (PRISM): a pilot study of a novel resilience-promoting intervention for adolescents and young adults with serous illness. *manuscript submitted*.
29. Rosenberg A, Yi-Frazier JP, Wharton W, Crouch JD, Wolfe J, Syrjala KL, Baker KS. Developing Resilience Interventions for Adolescents and Young Adults with Cancer and their Families. Paper presented at: Kathleen Foley Palliative Care Research Retreat2013; San Francisco, CA.
30. Rosenberg AR, Yi-Frazier JP, Eaton L, et al. Promoting Resilience in Stress Management: A Pilot Study of a Novel Resilience-Promoting Intervention for Adolescents and Young Adults With Serious Illness. *Journal of pediatric psychology.* 2015.
31. Connor KM, Davidson JR. Development of a new resilience scale: the Connor-Davidson Resilience Scale (CD-RISC). *Depress Anxiety.* 2003;18(2):76-82.
32. Campbell-Sills L, Cohan SL, Stein MB. Relationship of resilience to personality, coping, and psychiatric symptoms in young adults. *Behav Res Ther.* 2006;44(4):585-599.
33. Connor KM, Vaishnavi S, Davidson JR, Sheehan DV, Sheehan KH. Perceived stress in anxiety disorders and the general population: a study of the Sheehan stress vulnerability scale. *Psychiatry Res.* 2007;151(3):249-254.
34. Davidson JR, Stein, D. J. Rothbaum, B.O., Pedersen, R., Tien, X.W., Musgnung, J. Resilience as a predictor of remission in post-traumatic stress disorder. . US Psychiatric and Mental Health Congress; 2006; New Orleands, LA.
35. Bruwer B, Emsley R, Kidd M, Lochner C, Seedat S. Psychometric properties of the Multidimensional Scale of Perceived Social Support in youth. *Compr Psychiatry.* 2008;49(2):195-201.
36. Snyder CR, Harris C, Anderson JR, et al. The will and the ways: development and validation of an individual-differences measure of hope. *J Pers Soc Psychol.* 1991;60(4):570-585.
37. Irving LM, Snyder CR, Crowson JJ, Jr. Hope and coping with cancer by college women. *J Pers.* 1998;66(2):195-214.
38. Cohen S, Kamarck T, Mermelstein R. A global measure of perceived stress. *J Health Soc Behav.* 1983;24(4):385-396.
39. Strauss A, Corbin, J. *Basics of qaulitative reserach techniques and procedures for developing grounded theory.* Thousand Oaks, CA: Sage; 1998.
40. Tomich PL, Helgeson VS. Is finding something good in the bad always good? Benefit finding among women with breast cancer. *Health Psychol.* 2004;23(1):16-23.
41. Tomich PL, Helgeson VS. Five years later: a cross-sectional comparison of breast cancer survivors with healthy women. *Psychooncology.* 2002;11(2):154-169.
42. Michel G, Taylor N, Absolom K, Eiser C. Benefit finding in survivors of childhood cancer and their parents: further empirical support for the Benefit Finding Scale for Children. *Child Care Health Dev.* 2010;36(1):123-129.
43. Tomich PL, Helgeson VS, Nowak Vache EJ. Perceived growth and decline following breast cancer: a comparison to age-matched controls 5-years later. *Psychooncology.* 2005;14(12):1018-1029.
44. Kessler RC, Andrews G, Colpe LJ, et al. Short screening scales to monitor population prevalences and trends in non-specific psychological distress. *Psychol Med.* 2002;32(6):959-976.
45. Kessler RC, Green JG, Gruber MJ, et al. Screening for serious mental illness in the general population with the K6 screening scale: results from the WHO World Mental Health (WMH) survey initiative. *Int J Methods Psychiatr Res.* 2010;19 Suppl 1:4-22.
46. Furukawa TA, Kessler RC, Slade T, Andrews G. The performance of the K6 and K10 screening scales for psychological distress in the Australian National Survey of Mental Health and Well-Being. *Psychol Med.* 2003;33(2):357-362.
47. Green JG, Gruber MJ, Sampson NA, Zaslavsky AM, Kessler RC. Improving the K6 short scale to predict serious emotional disturbance in adolescents in the USA. *Int J Methods Psychiatr Res.* 2010;19 Suppl 1:23-35.
48. Kessler RC, Barker PR, Colpe LJ, et al. Screening for serious mental illness in the general population. *Arch Gen Psychiatry.* 2003;60(2):184-189.
49. Sherbourne CD, Stewart AL. The MOS social support survey. *Soc Sci Med.* 1991;32(6):705-714.

50. Heinonen H, Volin L, Uutela A, Zevon M, Barrick C, Ruutu T. Quality of life and factors related to perceived satisfaction with quality of life after allogeneic bone marrow transplantation. *Ann Hematol.* 2001;80(3):137-143.
51. Connelly JE, Wofford AB, Philbrick JT. Healthy patients who perceive poor health: why are they worried sick? *Am J Med Sci.* 2000;320(1):36-42.
52. Ware JE, Jr., Sherbourne CD. The MOS 36-item short-form health survey (SF-36). I. Conceptual framework and item selection. *Med Care.* 1992;30(6):473-483.
53. Prevention CfDCa. www.cdc.gov/brfss/.
54. Korn EL, Graubard BI. Epidemiologic studies utilizing surveys: accounting for the sampling design. *Am J Public Health.* 1991;81(9):1166-1173.
55. Nansel TR, Iannotti RJ, Liu A. Clinic-integrated behavioral intervention for families of youth with type 1 diabetes: randomized clinical trial. *Pediatrics.* 2012;124:e866-873.
56. Smith AW, Baum A, Wing RR. Stress and weight gain in parents of cancer patients. *Int J Obes (Lond).* 2005;29(2):244-250.
57. Wolfe J, Orellana L, Ullrich C, et al. Symptoms and Distress in Children With Advanced Cancer: Prospective Patient-Reported Outcomes From the PediQUEST Study. *Journal of clinical oncology : official journal of the American Society of Clinical Oncology.* 2015;33(17):1928-1935.
58. Kazak AE, Schneider S, Didonato S, Pai AL. Family psychosocial risk screening guided by the Pediatric Psychosocial Preventative Health Model (PPPHM) using the Psychosocial Assessment Tool (PAT). *Acta Oncol.* 2015;54(5):574-580.
59. Raudenbush SW, Bryk AS. *Hierarchical linear models : applications and data analysis methods.* 2nd ed. Thousand Oaks: Sage Publications; 2002.
60. Southwick SM, Bonanno GA, Masten AS, Panter-Brick C, Yehuda R. Resilience definitions, theory, and challenges: interdisciplinary perspectives. *Eur J Psychotraumatol.* 2014;5.
