# Supplementary material for: Effect of the Promoting Resilience in Stress Management Intervention for Parents of Children With Cancer (PRISM-P): A Randomized Clinical Trial
Source: JAMA Netw Open. 2019 Sep 18;2(9):e1911578. doi: 10.1001/jamanetworkopen.2019.11578 (PMC6751761; doi:10.1001/jamanetworkopen.2019.11578)
Supplement: Supplement 2. — eTable 1. PRISM Intervention Content Details eTable 2. Demographic Characteristics of Participants Who Did and Did Not Complete 3 Month Surveys eTable 3. Baseline Scores (Mean, SD) of Participants Who Did and Did Not Complete 3 Month Surveys [file jamanetwopen-2-e1911578-s002.pdf]

Supplementary Online Content

Rosenberg AR, Bradford MC, Junkins CC, et al. Effect of the Promoting Resilience in Stress Management intervention for parents of children with cancer (PRISM-P): a randomized clinical trial. *JAMA Netw Open*. 2019;2(9):e1911578. doi:10.1001/jamanetworkopen.2019.11578

eTable 1. PRISM Intervention Content Details

eTable 2. Demographic Characteristics of Participants Who Did and Did Not Complete 3 Month Surveys

eTable 3. Baseline Scores (Mean, SD) of Participants Who Did and Did Not Complete 3 Month Surveys

This supplementary material has been provided by the authors to give readers additional information about their work.

eTable 1. PRISM Intervention Content Details

Topic	Skills	1:1 Delivery	Group Delivery
Stress-management	Relaxation (deep breathing), Mindfulness (mediation), Identifying Social and Other Resources	In person or skype/phone, 30-60 minutes per session, approximately every 2-3 weeks, scheduled in tandem with planned hospital admissions or clinic visits.	In person (skype/phone available on individual request), half-day duration for all four sessions, scheduled approximately every other month based on participant availability. Goal 2-5 parents per group.
Goal-setting	Setting specific, measurable, actionable, realistic, time-dependent ("SMART") goals, planning for pitfalls and identifying alternatives		
Cognitive restructuring	Recognizing negative self-talk, replacing it with positive, neutral, or realistic perspectives		
Benefit-finding	Identifying gratitude, lessons, purpose, or meaning derived from experience of child's illness		
Boosters	Check-ins from staff inviting participants to practice skills-of-choice and/or share applied skills, accomplishments, and challenges.	In person/phone, once monthly	By email to whole group, once monthly

© 2019 Rosenberg AR et al. *JAMA Network Open*.

eTable 2. Demographic Characteristics of Participants Who Did and Did Not Complete 3 Month Surveys

	**Completed 3 month surveys (N=77)**	**Did not complete 3 month surveys (N=17)**
Study arm, n (%)		
UC	29 (38)	1 (6)
1:1	26 (34)	6 (35)
Group	22 (29)	10 (59)
Age in years, median (IQR)	37 (32, 42)	35 (32, 39)
Relationship to patient, n (%)		
Mother	59 (77)	14 (82)
Father	17 (22)	3 (18)
Other (Adoptive Grandmother)	1 (1)	0 (0)
Race categories, n (%)		
White	52 (68)	11 (65)
Asian	11 (14)	1 (6)
Black or African American	0 (0)	2 (12)
American Indian or Alaskan Native	3 (4)	0 (0)
Native Hawaiian or other Pacific Islander	5 (6)	0 (0)
Mixed/Other	5 (6)	3 (18)
No answer	1 (1)	0 (0)
Hispanic or Latino ethnicity, n (%)	4 (5)	2 (12)
First language English, n (%)	71 (92)	16 (94)
Highest Education, n (%)		
Less than high school	2 (3)	2 (12)
High School	18 (23)	5 (29)
College/Trade School	39 (51)	9 (53)
Graduate School	14 (18)	1 (6)
Other	4 (5)	0 (0)
Total combined family income for the past 12 months, n (%)		
Less than $25,000	10 (13)	6 (35)
$25,000 through $ 49,999	7 (9)	2 (12)
$50,000 through $ 99,000	26 (34)	4 (24)
$100,000 and greater	30 (39)	3 (18)
Don't know / prefer not to answer	4 (5)	2 (12)
Partner Status, n (%)		
Married	58 (75)	9 (53)
Not married, living with partner	6 (8)	1 (6)
No partner, never married	6 (8)	3 (18)
Divorced, separated, widowed	7 (9)	4 (24)
Cancer-type, n (%)		
Leukemia/Lymphoma	45 (58)	6 (35)
Central Nervous System (CNS) tumor	20 (26)	8 (47)
Non-CNS solid tumor	12 (16)	3 (18)

© 2019 Rosenberg AR et al. *JAMA Network Open*.

eTable 3. Baseline Scores (Mean, SD) of Participants Who Did and Did Not Complete 3 Month Surveys

Parent-Reported Outcome Measure	Completed 3 month surveys (N=77)	Did not complete 3 month surveys (N=17)
Resilience	28.8 (5.9)	26.5 (5.4)
Benefit Finding	51.5 (6.4)	47.9 (6.9)
Hope	3.5 (0.9)	3.2 (0.7)
Social Support	4.2 (0.8)	3.7 (1.1)
Physical functioning	91.8 (18.4)	77.5 (24.2)
Limitations due to physical health	72.3 (40.8)	56.3 (43.3)
Limitations due to emotional problems	52.2 (43.0)	52.9 (42.6)
Energy/fatigue	38.0 (20.9)	31.6 (17.0)
Emotional well-being	56.9 (19.1)	56.5 (16.8)
Social functioning	59.9 (29.0)	50.0 (26.5)
Pain	80.4 (18.9)	66.2 (30.5)
General health	66.3 (19.2)	59.1 (13.9)
Perceived Stress	21.7 (5.8)	23.2 (5.5)
Distress	8.9 (4.5)	8.8 (4.1)

© 2019 Rosenberg AR et al. *JAMA Network Open*.
