# Supplementary material for: Effect of the Promoting Resilience in Stress Management Intervention for Parents of Children With Cancer (PRISM-P): A Randomized Clinical Trial
Source: JAMA Netw Open. 2019 Sep 18;2(9):e1911578. doi: 10.1001/jamanetworkopen.2019.11578 (PMC6751761; doi:10.1001/jamanetworkopen.2019.11578)
Supplement: Supplement 3. — Data Sharing Statement [file jamanetwopen-2-e1911578-s003.pdf]

Data Sharing Statement

Rosenberg. Effect of the Promoting Resilience in Stress Management Intervention for Parents of Children With Cancer (PRISM-P). *JAMA Netw Open*. Published September 18, 2019. 10.1001/jamanetworkopen.2019.11578

Data
Data available: No

Additional Information
Explanation for why data not available: These data include protected information about the children of participants and thus will be kept confidential.
